# Can a Brief Interaction With Online, Digital Art Improve Wellbeing? A Comparative Study of the Impact of Online Art and Culture Presentations on Mood, State-Anxiety, Subjective Wellbeing, and Loneliness

**DOI:** 10.3389/fpsyg.2022.782033

**Published:** 2022-06-30

**Authors:** MacKenzie D. Trupp, Giacomo Bignardi, Kirren Chana, Eva Specker, Matthew Pelowski

**Affiliations:** ^1^Department of Cognition, Emotion, and Methods in Psychology, Faculty of Psychology, University of Vienna, Vienna, Austria; ^2^Max Planck School of Cognition, Leipzig, Germany; ^3^Department of Language and Genetics, Max Planck Institute for Psycholinguistics, Nijmegen, Netherlands; ^4^Vienna Cognitive Science Hub, University of Vienna, Vienna, Austria

**Keywords:** cultural engagement, receptive art engagement, wellbeing, mental health, digital art, art viewing

## Abstract

When experienced in-person, engagement with art has been associated—in a growing body of evidence—with positive outcomes in wellbeing and mental health. This represents an exciting new field for psychology, curation, and health interventions, suggesting a widely-accessible, cost-effective, and non-pharmaceutical means of regulating factors such as mood or anxiety. However, can similar impacts be found with online presentations? If so, this would open up positive outcomes to an even-wider population—a trend accelerating due to the current COVID-19 pandemic. Despite its promise, this question, and the underlying mechanisms of art interventions and impacts, has largely not been explored. Participants (*N* = 84) were asked to engage with one of two online exhibitions from Google Arts and Culture (a Monet painting or a similarly-formatted display of Japanese culinary traditions). With just 1–2 min exposure, both improved negative mood, state-anxiety, loneliness, and wellbeing. Stepdown analysis suggested the changes can be explained primarily via negative mood, while improvements in mood correlated with aesthetic appraisals and cognitive-emotional experience of the exhibition. However, no difference was found between exhibitions. We discuss the findings in terms of applications and targets for future research.

## Introduction

With the spread of the novel Coronavirus around early 2020, governments asked their citizens to stay at home to slow the rate of infection and protect the most vulnerable, forcing large parts of society to close their doors and everyday activities to move online. This included engagement with art and cultural institutions, which greatly increased online access to collections to connect with people now stuck at home (Radermecker, 2020; Samaroudi et al., 2020). Virtual tours emerged, encouraging people to “visit the museum from their couches” (Romano, 2020) and, for the first time, engaging with art and culture online was on the minds of a broad audience (Unitt, 2020).

At the same time, many online formats of art and culture signaled their potential application for a pragmatic purpose—ameliorating the negative pandemic-related effects on wellbeing and mental health. A number of projects and discussion in the press were built around suggestions that a brief engagement with a few works of art, or a trip to a virtual museum, might help soothe anxiety, boost one’s mood (Barbican Center, 2020), provide better wellbeing (International Arts and Mind Lab, 2020), or even help those isolated and lonely (Belvedere Museum, 2020). These aims seem to have been well received by the public, especially by those with low wellbeing and mental health issues, with evidence suggesting that a notable portion of individuals were, for the first time, seeking out online art encounters (Bu et al., 2021).

These developments are not unique. Rather, the present world-wide pandemic accelerated two trends that have come to define the arts’ pragmatic application for wellbeing and health: (1) Researchers and practitioners have considered art as a powerful tool to positively modulate wellbeing, which is widely-accessible, cost-effective, and non-pharmaceutical. In a growing body of evidence, when experienced in-person, engagements with art have been associated with positive outcomes (Fancourt and Finn, 2019 for review). (2) With the ubiquity of the internet, it is being increasingly recognized that digital, online formats might reach an even wider audience and present new possibilities for health-related use. If online engagement could provide a systematic impact, this would represent an even more cost-effective means of reaching large numbers in society (Clayton and Potter, 2017; Thomson et al., 2018), especially individuals who cannot visit museums, bringing doses of art into homes, hospital beds, or places of work without installation of a costly artwork. As put by one museum director (Gilman, in Rice, 2020), summarizing these dual trends changing the role of the arts, “right now, art… [is] more important than ever. It is one of the key things sustaining us while we are cocooning at home,” especially in its new online presence, “…it is what will nourish us as we adjust to the new normal of a post-COVID-19 world.”

However, despite this promise, this also raises several questions that have not seen much empirical research: *Can art or cultural engagements actually impact mental states such as wellbeing, state-anxiety, mood, satisfaction with life, or loneliness?* If so, *in which ways* and *by how much?* An answer to these questions has immediate pragmatic importance and raises important issues about the role and nature of typical art interventions, the potential importance of “real” artworks, and which factors might be key when considering impacts on wellbeing. Similarly, especially considering the wealth of *other* interventions that might be found online—*how does online art stack up against similar, non-art cultural engagements, such as other materials that might typically be found in museums and are similarly moving to the digital realm?* Is there anything particularly different about engaging with visual art? Further, this topic raises several tangential questions of interest, regarding how the nature of the art experience, and appraisal of an online exhibition might maximize or unlock specific wellbeing outcomes.

This is the aim of the present paper. Employing a quasi-randomized design in which individuals, via the internet, engaged briefly with art content (impressionist painting), we explore the potential impact on a number of factors—wellbeing (mood, life satisfaction, and subjective wellbeing), state anxiety, and loneliness—measured before and after viewing. We compare this intervention to another type of online cultural content (a display of Japanese culinary traditions), which employed a similar presentation format, but was generally expected to be seen as “not art” by participants and aligning more with receptive engagement such as visiting a history or science museum. We also consider secondary topics in an exploratory analysis, such as the nature of the experience at the appraisal and cognitive-emotional level, individuals’ label of the stimuli as “art,” and the interrelation of dependent wellbeing variables.

## Background—(Online) Art as a Wellbeing Intervention

The argument that art, in an online format, *could be* a tool for addressing wellbeing and mental health can be traced through past research; as can many outstanding questions.

Engagement with a variety of forms of arts and culture—*in person*—is now well-documented to support wellbeing and mental health. A 2019 World Health Organization review, included, for example, results from more than 900 publications (Fancourt and Finn, 2019) suggesting that engaging with various artistic or cultural activities can lead to meaningful impacts both in regards to preventative capacities (maintaining physical and mental wellbeing) and in support of a wide range of psychological issues. Among the many possible receptive or creative interventions (attending performances, making or viewing visual art or music, etc.), visually engaging artwork or visiting museums and galleries have increasingly been employed in partnership with public health initiatives, and healthcare providers in several countries have started to prescribe such activities as psychological health interventions (Packer and Bond, 2010; Camic and Chatterjee, 2013; Chatterjee and Noble, 2017; Todd et al., 2017; Thomson et al., 2018).

Among the various effects, empirical evidence has shown that on-site art interactions have been particularly associated with decreased loneliness by providing museums as spaces to learn and socially engage. This type of sustained engagement with institutions like art museums over the life span is associated with feeling less lonely and having greater levels of eudaimonic wellbeing (Todd et al., 2017; Tymoszuk et al., 2019, 2020). Further, participation in arts and cultural leisure activities was associated in cross-sectional studies with greater life-satisfaction, and mental health in a cohort of 8200 Norwegian adolescents (symptoms of anxiety and depression; Roberts et al., 2011; Hansen et al., 2015; Clayton and Potter, 2017). In many studies, the impact on mood and subjective wellbeing has also been illustrated. For example, in paradigms eliminating the activity of visiting a museum, Nanda et al. (2011) showed that simply hanging paintings of restorative nature scenes in common rooms led psychiatric patients to have reduced agitation (as reported by nurses) and need for anxiety medication compared to days without the paintings. Similar studies of art in patient or common rooms have been found to improve the anxiety, stress levels, depression, mood, and general wellbeing of both patients and staff. Further, the addition of a contemporary art gallery or bringing art to the patient’s bedside in a hospital, allows staff, patients and family members the ability to access art in difficult times and can lead to better mood and subjective wellbeing (Binnie, 2010; Roberts et al., 2011; Karnik et al., 2014; Hansen et al., 2015; Ho et al., 2015; Bennington et al., 2016; Davies et al., 2016; Wang et al., 2020). In regard to stress and anxiety, similar to the interventions that will be explored in this paper, Clow and Fredhoi (2006) asked individuals to take a 35-min visit to an art gallery on their lunch break and found that even short exposures lead to significantly lower self-reported stress (∼2.4 points on a pre-/post-visit 10-point scale) and cortisol concentrations. Impacts have also been found, see also Kweon et al. (2008), for similar paradigms showing lower reported stress following art installations in an office. Overall, there is a wealth of evidence that in-person art engagement can impact aspects of wellbeing including, subjective wellbeing, mood, anxiety, loneliness, and satisfaction with life.

The explanations of such impacts are themselves under debate (Mastandrea et al., 2019). However, they have been suggested to involve enjoyable or pleasurable experiences (Sachs et al., 2015; Chatterjee and Noble, 2017; Fancourt et al., 2020), which might improve aspects of subjective wellbeing by regulating mood. If this were the case, one might expect that the more enjoyable the experience with art is, the better improvement to aspects of wellbeing. They may be tied to shared social, communal factors (Roberts et al., 2011; Cuypers et al., 2012), escapism or removal from daily routine (see Kaplan, 1995), or even experiences of beauty as part of an aesthetic experience (Fancourt and Finn, 2019; Mastandrea et al., 2019). However, such aspects have received little attention systematically and need further research to examine which parts of art experiences are particularly important to impact wellbeing.

### Digital, Online Art—Would This Translate to Similar Wellbeing Impacts?

Online and digital technology *might* offer a natural ability to further empower the use of the arts in health and wellbeing formats. Today, art viewing, cultural engagements, and trips to art or other types of museums can take place via computers, tablets, smartphones and even virtual reality. This would certainly make it possible for many more individuals to engage art (Leng et al., 2014; Bu et al., 2021). Personal media, bringing artworks directly to a viewer could perhaps duplicate many of the same results, especially if beneficial effects are tied to the visual or cognitive-emotional aspects of experience. The potential for targeted, bite-size interactions with augmented information, seen in the comfort of one’s personal space without need for extra effort or distractions, perhaps could overcome some of the issues often given for why individuals may not enjoy art (Pelowski et al., 2014), allowing art to be more accessible, understandable, personal, even increasing impact beyond traditional installations or museum visits (Alelis et al., 2015).

At the same time, a long-running argument in especially art-critical, and more recently, psychological discussions suggests that art, for its full effect, might require to be seen in person, and that digital formats or other reproductions lose necessary aspects—immediacy, ambiance, level of engagement or importance, even artwork size—of the experience (Benjamin, 1968; Berger, 2008; Pelowski et al., 2017a; Specker et al., 2021). A handful of studies have suggested that art especially in digital formats, when compared to in-person gallery viewing, may lead to lower ratings of pleasantness (Locher et al., 1999, 2001; Locher and Dolese, 2004), interest (Locher et al., 2001; Locher and Dolese, 2004), liking, time spent viewing (Brieber et al., 2014) or even positive emotion or arousal (Brieber et al., 2015), all of which might be important for wellbeing benefits. Beyond this, if art impacts are driven more by the tangential aspects of an in-person visit—making a special trip, being with other people; even taking a break from everyday life activity or physical exertion itself (McLean, 2011)—these might be lost or diminished in online formats, or may connect to different results, for example, with factors such as life satisfaction or loneliness.^1^

The ability for online engagements to lead to wellbeing-related impacts especially requires further research. To our knowledge, only two studies have begun to provide tentative evidence. Leng et al. (2014) compared cultural activities (cooking and craft exercises) via tablets to traditional in-person activities, finding that participants displayed similar or better levels of wellbeing in the tablet group. Tyack et al. (2017) actually examined if art interventions could be delivered through touchscreen tablets. Assessing dementia patients in their homes; wellbeing (happiness, wellness and interestedness, 1–100 scale) was measured before and after freely viewing a set of artworks (with the ability to select and move between styles) via a specialized app designed for the study. The results showed that after each session of around 20 min, wellbeing increased, although not significantly. These papers conducted their studies with specialized samples of dementia patients, and as part of therapy programs (see also Zubala et al., 2021 for research on digital creative art therapy). Even in-person interventions have, somewhat surprisingly, not typically considered art brought into individuals’ homes. However, an ongoing mental health survey, conducted by University College London in the United Kingdom (Covid-19 Social Study, 2020), found a fifth of 70,000 respondents engaged more with the arts during lockdown than before. Those individuals who identified as having a mental illness or disability were likely to engage more in the first 22 weeks, which was speculated to be due to the move of cultural institutions online (Bu et al., 2021), however, this study did not quantify whether such use translated to actual effects.

### “Art” Versus “Non-art” Cultural Engagements?

The above issues touch another aspect that we explore in this paper, regarding how engagements with art might impact individuals via online formats in comparison to other varieties of cultural engagements. On one hand, engaging with “art” might be an ideal means to induce pleasurable and enjoyable experiences (Pelowski et al., 2017b). Previous research has found that viewing objects that one believes to be “art” (either via external cues or labels or derived from personal opinion) can lead to higher liking, beauty, pleasure (Locher et al., 2001; Leder et al., 2004; Pelowski et al., 2017b), positive affective experiences (Cupchik et al., 2009; Kirk et al., 2020), and even elicit greater activation of reward-related brain areas (Kirk et al., 2009; Lacey et al., 2011), all of which may lead to greater wellbeing or mood benefits (Berridge and Kringelbach, 2011; Becker et al., 2019). Having stronger aesthetic experiences, which is noted as a possible wellbeing mechanism (Fancourt and Finn, 2019), are likely to be particularly pronounced with art. This may be due to the perspective that one takes when engaging with something that they believe to be art. It is well known that such aesthetic perspectives are more evaluative, appreciative and contemplative of an object (Cupchik et al., 2009), which may specifically be the state that can enhance wellbeing. If the act of stopping to admire is important in wellbeing interventions, as supported by a study by Martínez-Martí et al. (2018) that found that learning to stop and admire beauty was effective in improving wellbeing, art could be much better than another type of engagement as it is, philosophy aside, there to be looked at, contemplated and admired.

On the other hand, wellbeing impacts can be tied to many other types of cultural engagement. For example, the in-person research referenced above is inclusive of a broad range of, often overlapping, cultural activities (see Davies et al., 2016). In just museum visits, one can find, among others, history, or science museums, which have been linked to wellbeing (Wheatley and Bickerton, 2017, 2019). Research has found positive impacts from the simple presence of other visual materials such as photos of nature (White et al., 2010). However, most research to date has not considered the impact of art in direct comparison to other types of cultural engagement in terms of health and wellbeing, and such distinction and comparison is argued to be a target for research (Ander et al., 2013). This is especially true with the internet, where there is a wealth of activities that offer potential wellbeing benefits (e.g., Son and Lee, 2021 for a recent study on stress and online shopping).

### Summary of Research Questions

To summarize, our main research questions include: How does art and cultural engagements impact mental states such as wellbeing, state-anxiety, mood, satisfaction with life, and loneliness? Second, how does online art compare to a similar, non-art online cultural engagement?

In addition, we examine the importance of several other dependent and independent factors in assessing the impact of online art, each of which, we address in our results section as *post hoc* and exploratory analysis.

First, we address if there are specific relationships between the different aspects of wellbeing outcomes (i.e., mood, anxiety, loneliness), which can also help to define the mechanisms underlying interventions. For example, past research has suggested that some aspects such as mood or state-anxiety may be particularly variable to immediate fluctuations in the environment, like an art intervention (e.g., Fredrickson and Branigan, 2005). These factors may then subsequently contribute to changes in more complex cognitive states such as loneliness and evaluative aspects of subjective wellbeing or life satisfaction and call for consideration.

Further, we explore how outcomes can depend on differing aspects of the individual’s experience. Research suggests that individuals’ reactions to art, and likely all forms of culture, are quite subjective (Pelowski et al., 2017b). Most basically, regardless of what kind of cultural engagement, this involves appraisal aspects such as whether one would like to visit again, finds beautiful or meaningful what they are engaging with.

Similarly, research has shown that—at the emotional and cognitive level—individuals respond differently to the same artwork. For example, the extent to which an engagement with art is more or less intellectually stimulating, educational, novel, harmonious, or boring, among other felt emotions (Pelowski et al., 2017c for review). Here we examine how these factors also differentiate impacts to wellbeing.

## Materials and Methods

### Participants

To test the above questions, the study included a final sample of 84 participants (65 women, 17 men, 1 other, 1 unknown; *M*_*age*_ = 34.89, *SD* = 14.47, range = 21–74). Participants were further divided between two conditions of cultural engagement, with 40 individuals (31 women, *M*_*age*_ = 35.40, *SD* = 14.44, range = 22–74) exposed to an “art” condition (see below) and 44 (34 women, *M_*age*_* = 34.43, *SD* = 14.64, range = 21–72) exposed to a “non-art” condition. Participants were recruited as a convenience sample through advertisements on social media and consisted of nationals from Europe (43%), the Americas (48%), Asia (8%), and Africa (1%). They received no compensation for participation. They engaged in the study between April 17 to June 6, 2020 during the first wave of the COVID-19 pandemic when most nations had instituted various degrees of lockdown.

The final sample was derived from an initial sample of 143, with participants removed due to exclusion criteria (see section “Results”). The study followed the ethical standards of the Declaration of Helsinki and the University of Vienna ethics board.

### Stimuli

**FIGURE 1:**
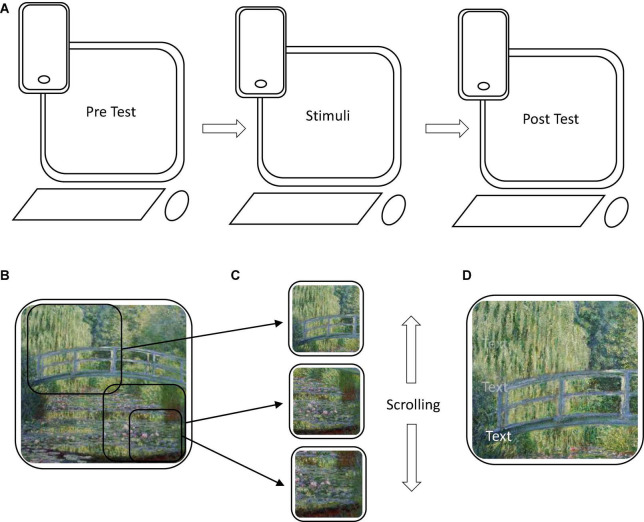
Study design, stimuli composition. **(A)** The procedure was a pre-post design which could be taken on both personal computers and smartphones. **(B)** Demonstration of stimuli showing “Waterlilies condition” as an example depicting Claude Monet, The Water-Lily Pond, 1899. (Oil on canvas, 88.3 × 93.1 cm. public domain image, Wikimedia commons). Stimuli from Google Arts and Culture were interactive, where smaller compositions of one main image **(B)** could be scrolled through **(C)** with accompanying text that faded away when scrolling down **(D)**. Available from: https://commons.wikimedia.org/wiki/File:Water-Lilies-and-Japanese-Bridge-(1897-1899)-Monet.jpg.

The online cultural engagement conditions consisted of two interactive exhibitions, broadly argued to be generally thought of as “art” and “non-art,” counterbalanced between subjects.^2^ See Figure 1 for stimuli examples and Supplementary Materials for an access link and all accompanying texts. Both sets of stimuli were selected from Google Arts and Culture, a free online repository of virtual art galleries, interactive experiences, and educational materials on a range of topics covering culture, history, and art (About Google Cultural Institute, 2020), and consisted of a single visual image which could be appreciated by itself, as well as zooming in on various details accessed by scrolling with the mouse. Though the order of details was predetermined, the participants had the autonomy to scroll up and down as they liked. Each detail was accompanied by related text (e.g., “…This is one of 18 canvases of this view in differing light conditions…”). Thus, in both cases they were aimed at duplicating what might be an engagement with a single painting or exhibit, where one might move forward or backwards and read accompanying labels or materials.

For the “art” condition (hereafter, “Water-lilies condition”), we used an exhibition of *Monet’s The Water-Lily Pond; An in-painting tour from the National Gallery, London* (Google Cultural Institute, 2020). This displays the single painting (*The Water Lily Pond*, 1899) of a bridge over a water-lily-festooned pond in Giverny, and was chosen because landscape and waterscape artworks have been suggested to be especially useful to reduce stress and anxiety among both patient and non-patient populations (Ulrich and Gilpin, 2003). The accompanying text in each frame focused on painting features (color, brushstrokes) or contextual information about the artist and painting.

The “non-art” stimulus (hereafter, “Bento condition”) was *A Bitesize History of Japanese Food; Explore a mouthwatering box of Japan’s iconic cuisine* (Google Cultural Institute, 2020). This explored a diagram in the shape of a bento box, containing photos and facts introducing the viewer to the history and traditions of Japanese food, and included images of food and food-related activities, such as harvesting or drinking. The text covered aspects of Japanese culture, including origins of specific dishes, agricultural traditions, and food preparation.

### Procedure

The study employed a mixed Two (Time: pre vs. post, within-participant) × Two (Online Cultural Engagement: art vs. non-art, between-participant) design. Participants were supplied with a survey link via Qualtrics, which led them to a short description of the study. The description noted that the study was designed to test participants before and after they freely explored an online exhibit, and the types of experiences that could be engendered. After agreeing to participate, participants were first asked to provide demographic information, and then completed a pre-viewing baseline measure of our dependent variables. The order of the scales for both the pre-viewing baseline and the post test was randomized for each participant to avoid any sequence bias. Upon beginning this, participants were also already assigned, unbeknownst to them, to one of the two types of online engagements, with the general description being the same for both the Water-lilies and Bento conditions. In both cases, we refrained from using the term “art” but rather referred to an “online experience” or “exhibit” in all instructions. Participants were explicitly instructed not to enter any other parts of the website or other webpages and to return to the post survey once finished. They were then presented with a hyperlink button which, upon clicking, would open either the art or non-art Google Arts and Culture exhibition in a new window, where they were able to spend as much time as they liked viewing and interacting with the stimuli.

### Pre-viewing Survey – Demographics and Personality, and COVID Related Conditions

In the pre-study survey, participants provided demographic descriptions (age, gender, nationality, location of current residence) as well as the status of their lives during the present lockdown, including lockdown severity (ranging from “I am staying only in my personal residence” to “I am not in lockdown at all,” see Supplementary Table 1), duration, and number of other people living in the participant’s residence. The Ten Item Personality Inventory (TIPI) was used to assess personality traits (Gosling et al., 2003). Art expertise was assessed through four items derived from the Vienna Art Interest and Art Knowledge Questions (Specker et al., 2020) on art education or training, art interest, and art related behavior. We also asked if participants had ever visited any online art presentation previously in their lives.

### Pre-/Post-viewing Survey—Wellbeing, Anxiety, Mood, Loneliness

To measure the impact of visiting the online material, we assessed six dependent variables (hereafter “Wellbeing DVs”). These were presented in random order in both the pre- and post-viewing survey to assess change after the brief online engagement; (1) The De Jong Gierveld 6-Item Loneliness Scale (De Jong Gierveld and Van Tilburg, 2010); (2) State-Trait Anxiety Inventory (Marteau and Bekker, 1992); (3) The Satisfaction with Life scale (Diener et al., 1985); (4) The Subjective Wellbeing scale (Tinkler and Hicks, 2011); (5–6) Mood was assessed by two questions asking participants to rate their overall positive and negative mood. Note, all Likert-type scale-based questions here and in the post-study were adapted to seven points to aid consistency of answering.

### Online Experience Evaluations

In the post-viewing questionnaire, participants also appraised the stimuli via four questions on a 7-point scale (1 = ‘not at all,’ 7 = ‘very much’), indicating how beautiful, meaningful, and good the stimuli were, and how much they would like to visit the website again. Participants were also asked if they felt they had seen ‘art’ during the experience or not (binary forced-choice: “I saw art”/“I saw something else”).

As an exploratory means of collecting more information on the nature of the experience, participants were presented with a list of 55 cognitive-emotional terms (e.g., “serenity,” “bored,” “angry,” and “harmonious,” etc.), and asked to express how much they felt each of them while they were viewing the stimulus (1 = ‘not at all,’ 7 = ‘very much’). See Supplementary Table 2 for full list. Previously, variations of this list have been used to further examine types of art experiences (Pelowski, 2015; Pelowski et al., 2018, 2019).

## Results

Participants were included in the final sample if they had completed more than 65% of the total survey, allowing at least one pairwise comparison, and if they had spent at least 10 s viewing the online materials. This cut off was selected based on the mode viewing time reported in a classic study of art viewing by Smith et al. (2016) which was 10 s, also consistent with other studies (see for review Smith and Smith, 2001; Pelowski et al., 2017c; Verhavert et al., 2018). Forty participants were excluded due to insufficient survey completion; 19 were excluded due to overly short viewing times. A histogram of the time spent viewing can be found in the Supplementary Figure 1.

Supplementary Table 1 provides the demographic and art interest descriptive statistics across the final sample of participants divided between conditions, as well as between-conditions statistical comparisons. A comparison of individual’s average art interest, typical art engagement, and art training found no significant differences between groups (Supplementary Table 1). In addition, there were no significant differences in days spent in lockdown (*M* = 50.6), age, or personality.

Here, and throughout the results section, we employed a mix of parametric and equivalent non-parametric analyses on a case-by-case basis, depending on violation of assumptions. These are indicated where applicable.

### Descriptive Results of Viewing Time, Appraisal and Experience of Online Engagements

**FIGURE 2:**
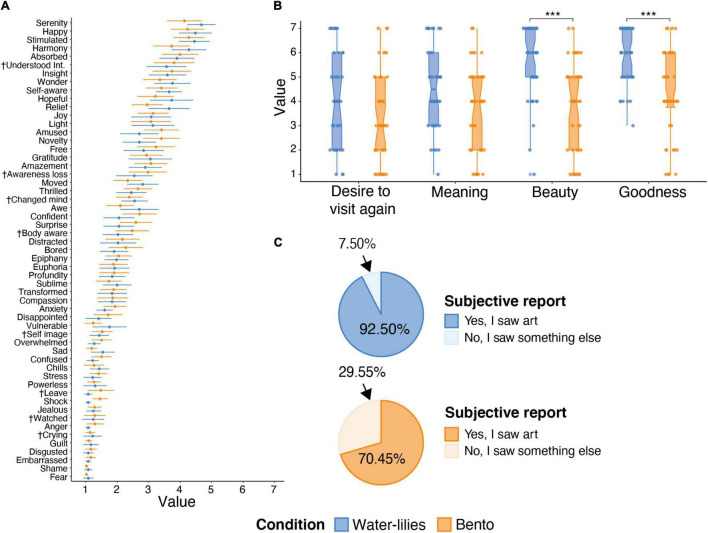
**(A)** Shows means and 95% CI intervals for participants ratings of cognitive-emotional states during the online cultural engagements for the Water-lilies (light blue) and Bento (light orange) conditions.^†^ shorthand labels, see Supplementary Material for the complete labels. **(B)** Box plots showing the ratings for aesthetic evaluation for the Water-lilies and Bento conditions, showing that in general Water-lilies condition was rated more highly. **(C)** Pie chart showing percentages of participants who reported to see art or something else while engaging online with the Water-lilies and Bento conditions.

Mean results of reported appraisal and cognitive-emotional terms are shown in Figure 2 (see also Supplementary Table 2 for descriptive statistics). Comparison between conditions for whether individuals believed they had seen a work of “art” versus “something else” revealed, as expected, a significant difference [χ^2^ (1,84) = 5.25, *p* = 0.02], with 92% of individuals in the Water-lilies condition agreeing they saw art, compared to 70% in the Bento condition (this, a still rather high result, is also considered further below).

Participants in both conditions, on average, evaluated their online engagements positively, with Wilcoxon rank-sum tests conducted for each variable independently revealing comparatively higher beautifulness and goodness appraisals in the Water-lilies condition (*Z* = −4.18, *p* < 0.001, and *Z* = −4.56, *p* > 0.001), whereas meaningful and desire to visit again did not show differences (*Z* = −1.81, *p* = 0.279 and *Z* = −2.06, *p* = 0.157, respectively; *p*-values adjusted with a Bonferroni correction for multiple comparisons; Non-parametric tests employed due to violation of normality of appraisals’ distributions, see Supplementary Figure 4). The four appraisals themselves also showed positive intercorrelation (*r*_*s*_ = 0.55–0.71) across conditions.

On average, participants engaged with the online content for around one-and-a-half to two minutes (Water-lilies: *M* = 107 s, *SD* = 69 s, range = 11–274 s; Bento: *M* = 209 s, *SD* = 249 s, range = 10–1401 s). A Wilcoxon rank-sum test comparing conditions was not significant (*Z* = −1.76, *p* = 0.08). Across conditions, viewing time was also not correlated with the four appraisals of the material themselves (all *r*_*s*_ = 0.04–0.1, scatterplots of relationship between time spend and each DV per condition can be found in Supplementary Figure 3).

When asked how they felt while engaging the online content, participants indicated quite similar positive-valence emotions and cognitive states (e.g., serenity, happy, stimulated, insight), with low levels of negative emotions (fear, embarrassed, anger, etc.), in both conditions (see Figure 2 for comparisons at the individual item level).

### What Is the Impact of Art and Non-art Online Engagements on Wellbeing and How Do They Compare?

**FIGURE 3:**
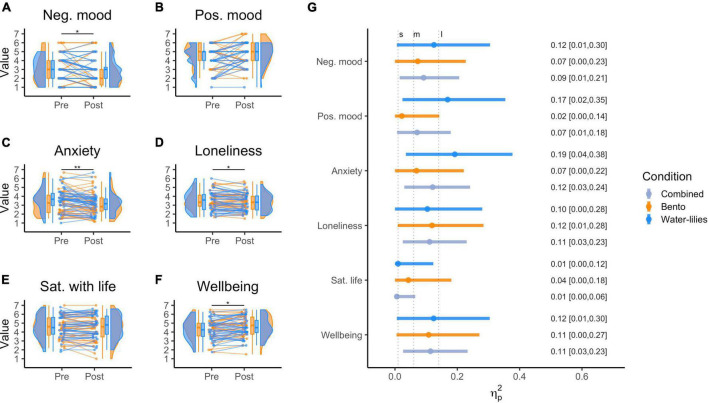
**(A–F)** Raincloud plots showing the distributions of rating pre and post online cultural experience grouped by condition. The boxplots display the median (horizontal line middle of box), the data within the 25th and the 75th percentile (inside box), and the data within the 10th to the 25th and the 75th to the 90th percentile (vertical line); Dots represent individual ratings; Slopes represent changes between pre and post. Images were computed following Langen’s tutorial (Allen et al., 2021; see https://github.com/jorvlan/raincloudplots); **(G)** forest plot of the effect sizes (partial eta square) of the online experience on the six DVs. Vertical dashed lines are used to indicate intervals to interpret the strength of the effect. S, M, L indicate small, medium, and large effect size intervals.

Descriptive statistics for the Wellbeing DVs, pre- and post-viewing, are provided in Table 1 and visualized as violin plots with individual slopes for each participant, in Figure 3. Effect sizes for each condition and the total sample are shown in Figure 3. Correlations between Wellbeing DVs (pre-, post-, and post-pre changes) are shown in Supplementary Figure 2.

**TABLE 1:** Average participant self-report ratings for each time condition per group (means and standard deviations).

Variable		Water-lilies*^a^*				Bento*^b^*			
		Pre		Post		Pre		Post	
Negative mood	*M* (*SD*)	3.08	(1.32)	2.75	(1.36)	2.95	(1.53)	2.64	(1.60)
Positive mood	*M* (*SD*)	4.31	(1.39)	4.75	(1.18)	4.69	(1.24)	4.86	(1.34)
Anxiety	*M* (*SD*)	3.60	(1.18)	3.19	(1.16)	3.31	(1.32)	3.09	(1.24)
Loneliness	*M* (*SD*)	3.49	(1.10)	3.32	(0.93)	3.50	(0.93)	3.34	(0.97)
Life satisfaction	*M* (*SD*)	4.54	(1.28)	4.95	(1.30)	4.52	(1.21)	4.41	(1.40)
Wellbeing	*M* (*SD*)	4.26	(1.12)	4.49	(1.11)	4.41	(1.14)	4.66	(1.14)

*N^a^ = 36, N^b^ = 42, (see exclusion criteria). For a positive impact to occur, negative mood, anxiety, and loneliness should decrease whereas positive mood, satisfaction with life and wellbeing should increase.*

As can be seen, in general, participants showed mean baseline scores (before viewing) at just below the midpoints for most DV scales, albeit with a rather large spread across participants. We also found a general moderate correlation between most baseline Wellbeing DVs (Supplementary Figure 2). No differences in baseline scores were found, for all Wellbeing DVs, when comparing between condition groups (Supplementary Table 1).

In general (Figure 3) the Wellbeing DVs, when considered after the online engagements, tended to show mean changes in the expected directions (i.e., decreased state-anxiety, loneliness, negative mood; increased positive mood and wellbeing), with the Water-lilies conditions tending to have generally larger effect sizes.

To statistically test the results, we first conducted a two-way mixed repeated measures multivariate analysis of variance (MANOVA) on the six Wellbeing DVs, with *Time* (pre, post; within-subjects) and engagement *Condition* (Water-lilies, Bento; between-subjects) as independent variables (conducted in Rstudio, R version 3.6.2; RStudio Team, 2019). In MANOVA, group differences are tested by creating a linear combination of the Wellbeing DVs that maximize group differences, and is considered to be optimal when Wellbeing DVs show correlation (Tabachnick and Fidell, 2013). This was conducted on a reduced sample of 78 participants (36 Water-lilies, 42 Bento) due to three participants having missing data and the detection of three multivariate outliers (2 Bento, 1 Water-lilies) with Mahalanobis distance exceeding the critical value (22.46; *df* = 6, MD1 = 23.35, MD2 = 24.52, MD3 = 24.81). Analyses including the outliers did not substantially differ, however, it impacted the marginal significance of a covariate in one of the *post hoc* analysis (see Table 2). The Wellbeing DVs were not multivariate normally distributed, however, MANOVA has been shown to be robust to violations of the assumption of multivariate normality when the sample is, as in our case, >30 per condition (Tabachnick and Fidell, 2013). The data met assumptions for homogeneity of variance (all Levene’s tests *p* > 0.05) and multicollinearity for Wellbeing DVs (correlations between Wellbeing DVs were < 0.8).

**TABLE 2:** Stepdown analysis statistics.

Variable	Analysis	Covariate	*Pre*	*Post*	*df*	*F*	*p*(*adj*)	$\eta_{p}^{2}$	90% CI	
			*M* (*SD*)	*M* (*SD*)					LL	UL
Negative mood	Univariate	–	3.01 (1.43)	2.69 (1.49)	1, 77	7.81	**0.039**	0.09	0.15	0.21
Positive mood	Univariate	–	4.51 (1.32)	4.81 (1.26)	1, 77	5.92	0.104	0.07	0.00	0.18
	Step-down	Negative mood			1, 76	49.72	**<0.001**	0.40	0.25	0.51
	Step-down				1, 76	0.83	0.353	0.01	0.00	0.08
Anxiety	Univariate	–	3.44 (1.26)	3.14 (1.20)	1, 77	5.92	**0.001**	0.12	0.03	0.24
	Step-down	Negative mood			1, 75	29.71	**<0.001**	0.28	0.15	0.41
	Step-down	Positive mood			1, 75	13.72	**<0.001**	0.15	0.05	0.28
	Step-down				1, 75	3.68	0.406	0.05	0.00	0.15
Loneliness	Univariate	–	3.50 (1.00)	3.33 (0.95)	1, 77	9.64	**0.016**	0.11	0.03	0.23
	Step-down	Negative mood*^a^*			1, 74	3.47	0.067	0.06	0.00	0.16
	Step-down	Positive mood			1, 74	1.26	0.265	0.02	0.00	0.09
	Step-down	Anxiety			1, 74	6.14	**0.016**	0.08	0.00	0.18
	Step-down				1, 74	4.54	0.219	0.05	0.00	0.14
Life satisfaction	Univariate	–	4.53 (1.24)	4.50 (1.35)	1, 77	0.00	>0.999	0.01	0.00	0.01
	Step-down	Negative mood			1, 73	0.01	0.932	0.01	0.00	0.07
	Step-down	Positive mood			1, 73	0.18	0.281	0.01	0.00	0.09
	Step-down	Anxiety			1, 73	0.14	0.705	0.00	0.00	0.01
	Step-down	Loneliness			1, 73	0.13	0.721	0.00	0.00	0.05
	Step-down				1, 73	0.53	0.353	0.00	0.00	0.05
Well-being	Univariate	–	4.34 (1.12)	4.58 (1.12)	1, 77	9.90	**0.014**	0.11	0.03	0.23
	Step-down	Negative mood			1, 72	6.19	**0.015**	0.08	0.01	0.19
	Step-down	Positive mood			1, 72	4.57	**0.036**	0.06	0.00	1.16
	Step-down	Anxiety			1, 72	1.31	0.256	0.00	0.00	0.06
	Step-down	Loneliness			1, 72	3.26	0.075	0.04	0.00	0.15
	Step-down	Life satisfaction			1, 72	0.30	0.588	0.02	0.00	0.10
	Step-down				1, 72	3.44	0.353	0.04	0.00	0.14

*CI, confidence interval; LL, lower limit; UL, upper limit. The bolded values indicate statistically significant values.*

*^a^Before exclusion of three multivariate outliers, negative mood explained a significant proportion of the change in loneliness, p = 0.032.*

The analysis showed a main effect of *Time* {*F*(6,71) = 3.65, *p* = 0.003; $\eta_{p}^{2}$ = 0.24, 90% CI = [0.05; 0.31]} across both conditions. However, neither the main effect of *Condition* [*F*(6,71) = 0.56, *p* = 0.763] nor the *Condition × Time* interaction [*F*(6,71) = 0.64, *p* = 0.700] significantly affected the linear combination of Wellbeing DVs. These results suggested that both types of online cultural engagement significantly impacted the combined Wellbeing DVs; however, there was not a significant difference between the impact of the Water-lilies and Bento conditions ($\eta_{p}^{2}$ = 0.05, 90% CI = [0; 0.07]). An additional series of repeated measures ANOVAs, run independently for each of the Wellbeing DVs and looking at potential differences between conditions, showed all interactions with *Time* not to be significant, with *p*-values ranging from *p* = 0.171 to *p* = 0.971 (all *p*-values uncorrected, see Supplementary Table 3).

### What Is the Impact of Online Engagement on Individual Wellbeing DVs?

To further consider the impact of the online interventions on the specific Wellbeing DVs which were assessed above, as well as their potential interrelation, we carried out a Roy-Bargman stepdown analysis. This approach is suggested as a best-practice follow up for MANOVA (Tabachnick and Fidell, 2013), especially in cases of correlation between Wellbeing DVs as noted above. This procedure consisted of, first, univariate post-hoc ANOVAs on each individual DV, in order to examine main effects of time at the individual DV level.

This was followed by a set of ANCOVAs, which tested for the effects of *Time* on each of the Wellbeing DVs, taking into account other Wellbeing DVs as covariates in a prioritized, step-down order. This second step allowed us to control for effects that are shared between the Wellbeing DVs, a phenomenon found in past studies looking at factors of wellbeing (i.e., Dua, 1993; Cohen et al., 2017). As a consequence of the above findings of a significant impact of the online engagements across both conditions with a non-significant difference between them, the samples were combined for these two subsequent analyses. All independent *p*-values for each of the main effects in the following tests were corrected for the number of comparisons (*N* = 6, *p*-values for covariates were not corrected).

At the univariate level, the results, reported in Table 2 (see also Figure 3 for effect sizes), indicated that the online cultural engagements had a significant impact on four of the Wellbeing DVs—*negative mood, state-anxiety, subjective wellbeing*, and *loneliness*—but not on positive mood or satisfaction with life.

For the stepdown analysis, the effects of the online engagement on the Wellbeing DVs were prioritized as follows: (1) effect on mood (negative and positive); (2) effect on negative variables (state-anxiety and loneliness), after having controlled for the effect of mood; and (3) the effect on positive wellbeing variables (satisfaction with life and subjective wellbeing) after accounting for changes in mood and negative variables. This order was selected because, as noted in the introduction, changes in mood and state-anxiety are argued to be potentially more variable as a result of changes in one’s immediate environment, which can possible contribute to changes in more complex cognitive states (Fredrickson and Branigan, 2005). Homogeneities of the regression slopes were obtained for all of the subsequent univariate analyses.

The results suggested that changes observed in state-anxiety and wellbeing were not uniquely impacted by the online cultural engagements beyond the shared effect on mood. For loneliness, although significantly impacted by the online engagements, the effect was accounted for by changes in state-anxiety, after controlling for the effect of changes in mood.

### Exploratory Analyses

We then turned to a *post hoc* exploratory analysis, considering some of the other research questions noted in this study, as well as some of the above-reported findings.

#### Do Appraisals of the Experience Relate to Impacts on Wellbeing?

**FIGURE 4:**
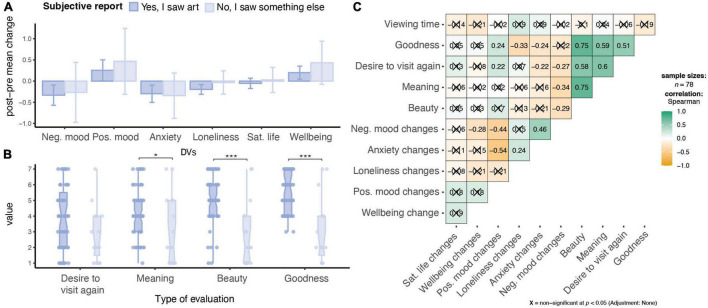
Subjective experience of participants, including **(A)** DV changes grouped by perception of if participants considered they say art or not, bar plots showing the breakdown of changes in Wellbeing DVs. Error bars represent 95% CI. **(B)** Box plots showing the breakdown of the aesthetic ratings grouped by subjective reports (i.e., seeing art), Error bars represent SD. *p < 0.05, ***p < 0.001. *p*-values uncorrected. **(B)** Appraisals shown broken down by I saw art or not. **(C)** Correlations between viewing time, appraisals and changes in Wellbeing DVs. Correlation plot was computed using ggstatplot (Patil, 2021).

First, as a general consideration of the relation between the nature of experience and its impact, we considered the correlation between appraisals of the online material (meaning, beauty, goodness, desire to visit again) and the change scores (post-pre) for the individual Wellbeing DVs. We similarly considered viewing time. Due to the violations of normality, we used Spearman’s Rank correlations. Results for combined online conditions are shown in Figure 4. Results split between conditions can be found in Supplementary Table 4.

Decreases especially in negative mood, as well as state-anxiety and positive mood, were significantly related to relatively higher appraisals. Changes in negative mood were related to reported desire to visit the experience again, meaningfulness, beauty, and marginally related to goodness. Similarly, changes in state-anxiety and positive mood were significantly related to desire to visit again, goodness, with state-anxiety also marginally related to beauty. Goodness alone also was correlated with lower loneliness. Note, however, that, only the relationships between meaningfulness and changes in negative mood and goodness and changes in loneliness remained significant or marginally significant (after multiple comparisons testing) (adjusted *p*_*s*_ = 0.049 and 0.067, respectively; Bonferroni correction for 24 tests). Amount of time spent by individuals was not significantly related with any DV change (*r_s_* = –0.21–0.19).

#### Does Seeing “Art” Influence Impact on Wellbeing?

We then followed up on participants’ classification of the stimuli as “art,” considering the large number of individuals that considered even the Bento condition to be an artwork. A breakdown between participants who said they saw either “art” or “something else,” regardless of their actual Water-lilies/Bento assignment, comparing appraisals of beauty, goodness, meaningfulness, and desire to visit again are shown in Supplementary Table 5. A breakdown of DV change scores for “I saw art” and “something else” is shown in Supplementary Table 6. Difference in appraisals and Wellbeing DVs are shown in Figure 4. To consider these distinctions, we used multivariate analysis with groups, “I saw art” group (*N* = 63) or “I did not see art” (*N* = 15). Wilcoxon rank sum test suggested that participants who indicated that they saw “art,” regardless of assigned condition, reported significantly higher meaningfulness (*Z* = −2.29, *p* = 0.022), beauty (*Z* = −3.72, *p* < 0.001), and goodness (*Z* = −3.99, *p* < 0.001), and marginally significant higher desire to visit again (*Z* = −1.91, *p* = 0.055).

However, a series of two-sample nonparametric Welch’s *t*-tests, comparing change scores between conditions, did not reveal significant differences between those who reported seeing “art” or “something else” for any DV (all *p*-values *>* 0.26).

#### Do Cognitive-Emotional Experience Factors Predict Impact on Wellbeing?

Finally, we returned to the list of the cognitive-emotional items that participants reported feeling during their experiences. To reduce the data and to identify potential patterns, we conducted a Principal Component Analysis (PCA) on all 55 items. A parallel analysis using Monte Carlo simulation with permutations (5000) of the raw data set, conducted in SPSS with the rawpar.sps script (O’Connor, 2000), suggested four significant components. These were then assessed using a Direct Oblimen rotation, which allows for an orthogonal or oblique solution, expected to provide a more natural fit for the data. The Kaiser–Meyer–Olkin measure verified sampling adequacy (KMO = 0.65; Field, 2009). Bartlett’s test of sphericity *X*^2^ = 3359.9, *p* < 0.001, also indicated sufficient correlations between items to support the PCA analysis.

The resulting rotated components solution (Supplementary Table 7) accounted for 48.48% of the variance, with a breakdown of items more or less in line with past findings (Pelowski, 2015; Pelowski et al., 2019). The first component (26.20% of variance) largely consisting of items related to positive or socially focused terms (highest loadings: confident, changed self-image, compassion, gratitude, free, sublime, etc.). The second component (11.11%) described largely negative and again socially focused terms (fear, like crying, sad, powerless, stress, shame, guilt, vulnerable). The third component (5.91%) included items previously suggested to indicate largely “facile” (Pelowski, 2015) or unrewarding experience (disappointed, bored, need to leave, embarrassed, and confused). The fourth component (5.30%) described more insightful or transformative aspects (absorbed, thrilled, insight, change mind, epiphany, etc.).

Component scores were then calculated per component for each participant (*via* regression method). These scores were then used in a series of multiple regressions to predict change in the Wellbeing DVs. See Supplementary Tables 8–10 for all results both considering combined or split between conditions. Once again, changes in especially negative mood emerged as being significantly predicted by the combined model [*F*(4,69) = 6.29, *p* < 0.001, *R*^2^ = 0.27]. Further analysis revealed that such changes were significantly driven by Component 1 (“*positive/social affect”*; *t* = −2.15, *p* = 0.035), with higher scores relating to a decrease in negative mood, and to lower scores of Component 3 (“*facile”*; *t* = 3.75, *p* > 0.001).

Decreasing state-anxiety was also significantly predicted, again, by component one [*t* = −2.39, *p* = 0.020; main model (*F*(4,69) = 2.285, *p* = 0.069, *R*^2^ = 0.12]. No other Wellbeing DVs were significantly predicted by any of the components (*p*-values are uncorrected). Interestingly, as with the appraisals above, the relationships between the cognitive-emotional components and Wellbeing DVs also tended to emerge more saliently in the Water-lilies condition, whereas with the Bento condition no significant state-anxiety or mood-related effects were found.

## Discussion

We found a significant impact on several wellbeing variables in a pre-/post-paradigm in which individuals were asked to briefly visit an online art exhibition (one Monet painting) or another similarly-formatted, “non-art” cultural engagement (a display of Japanese culinary tradition, aligning more to what might be encountered in a history museum offline). This included lowered state-anxiety, negative mood, loneliness, and increased subjective wellbeing. This occurred, in the art condition, with an interactive display of one painting, and in the other display of cultural material, and in both cases, over durations averaging one-and-a-half to two minutes, notably with no significant relationship between positive impact and actual time spent engaging.

A stepdown analysis found that the changes in Wellbeing dependent variables (Wellbeing DVs) tended to be explained by changes in negative mood (in the case of positive mood, state-anxiety and wellbeing), while improved loneliness was explained by improvements in state-anxiety. Finally, the “art” condition had generally larger effect sizes, but was not significantly ‘*better*’ than “non-art” at improving any Wellbeing DV. When considering the nature and appraisals of the experience, a significant relationship was found between decreases in negative mood, and to a lesser extent state-anxiety and positive mood, and positive appraisals of the experience (desire to visit again, beauty, and, most pronounced, meaningfulness). Similarly, changes in negative mood and state-anxiety related to higher reported positive or socially focused cognitive-emotional states (Component 1).

### General Efficacy for Online Art and Cultural Interventions

These results raise several implications for both pragmatic and theoretical application of the arts for wellbeing. First, this provides initial evidence that such wellbeing effects can be found via brief interactions with art and similar cultural materials online, impacting many of the same variables as considered in in-person studies. This finding, detected with an adult sample of convenience, during a unique COVID-19-induced lockdown period, might be viewed as proof of concept that technology, via the internet, can deliver targeted doses of art and culture into the everyday lives of individuals. The findings also contribute evidence to the potential of digital reproductions to produce similar effects to those found with real artworks installed in common spaces or seen in-museum visits.

The reduction of reported loneliness also merits specific mention. While probably only tangentially connected to the art experience—via the changes in anxiety—a claim could be made that the loneliness-reducing aspects of cultural engagements, routinely found in-person (Todd et al., 2017; Tymoszuk et al., 2019), might come about from the social connection or collaborative nature of visiting a museum or sharing art experiences. Here, however, we found that even visiting an exhibit, likely by oneself via the internet, led to significant effects. This is possibly due to the communal nature of visiting a public website and seeing another person’s art or design. In support of such a communal claim, loneliness was one of the only variables that showed slightly larger effects in the Bento condition, which focused on human activities in a more explicit way. This adds credence to recent initiatives, especially arising during COVID-19, for online arts and culture exhibitions to target loneliness during isolation from others—another compelling target for future research.

In both conditions we found lack of significant effects on positive mood and satisfaction with life. When considering satisfaction with life, this is likely due to the more global wellbeing nature of the construct, in that the questions are asking about a more overall assessment of one’s satisfaction, which is possibly not likely to change on short timescales. It would be interesting, however, to include more testing timepoints to address this in future study as satisfaction with life has been noted to be related to cultural engagement like visiting art museums (Lee et al., 2020). In regard to positive mood, it was slightly surprising to find a non-significant effect but at the same time to find a significant effect in the reduction of negative mood. It seems that both art and cultural engagement may be specifically better targeted at decreasing negative feelings while not as effective at increasing positive ones. Here it could be seen that visiting an online art and cultural website, like Google Arts and Culture, may help decrease feelings like sadness, anxiety or agitation but may not increase feelings of happiness, warmth, satisfaction. It would be especially valuable for future research to include a measurement of mood that has a variety of feeling items that can help to disentangle this further.

When comparing the effect of online art interventions to past literature, our findings are similar. Looking to the two most salient effects found in this paper—improvement in mood and state-anxiety—we found moderate to large effects in the art condition (negative mood $\eta_{p}^{2}$ = 0.12, positive mood $\eta_{p}^{2}$ = 0.17, and state-anxiety $\eta_{p}^{2}$ = 0.19). These are in line, for example with Ho et al. (2015) who reported a pre/post change (assessed via Brief Mood Introspection Scale) following an art exhibition visit in a hospital. As well as Paddon et al. (2014), who reported medium effect sizes in negative mood (Cohen’s *d* = 0.38) and positive mood (*d* = 0.69), assessed via Positive and Negative Affect Schedule (PANAS), following a cultural museum object-handling session in a hospital [also see Clow and Fredhoi’s (2006) report of reduction in self-reported stress after individuals spent a lunch break in-person in a museum of art].

Even comparing to other domains, our results remain roughly equivalent. For example, Cracknell et al. (2016) reported a change in affective valence (assessed via 11-point bipolar Feeling Scale), following a roughly 5-min intervention in which participants observed an aquarium fish tank, $\eta_{p}^{2}$ = 0.47 (negative-to-positive). Comparing across interactions with nature, which have been routinely shown to provide impacts on mood, a meta-analysis (McMahan and Estes, 2015) of both lab or in-person interventions reported a standardized reduction in negative affect across studies of *r* = 0.12 (increase in positive affect *r* = 0.31). In emerging results of other online interventions, Howells et al. (2015) report a smartphone-based mindfulness intervention impact on affect (measured via PANAS after 10 days of use) of medium size effect (η^2^ = 0.071) for positive affect and small effect (η^2^ = 0.010) for negative mood. Keeping in mind the self-report method of the present study, differences in assessment scales, and leaving open the question of the duration of impact or whether self-report might relate to physical or behavioral differences, our results provide compelling evidence for art and culture in online spaces.

### Is There Anything Different About Engaging Art Versus Other Cultural Content?

In regards to our second research question, we found mixed, albeit still compelling, suggestions about the potential role that “art” might play in improving wellbeing. We did not find a significant difference between conditions. One probable explanation for the lack of difference is that both conditions, which were selected to be formally similar, evoked similar responses—for example, a generally positive interaction with visually pleasing material. Note, while the artwork was rated as more beautiful and good, both conditions were equally meaningful and evoked a desire to experience them again.

One telling result in line with the above argument was that, despite explicitly not labeling it as such on our part, we found almost 70% of those in the Bento condition actually believed that they had engaged with “art.” This finding that individuals would label something—even if not intended to be seen as art in a classic sense—as an “art” example, is in line with past research. For example, Pelowski et al. (2017b) reported this with photographs of a number of random every-day objects, suggesting that individuals may use “art” as a general label of particularly appreciated visual stimuli. Thus, it is reasonable to assume that we were not in fact comparing apples to oranges as we had designed, but more likely that we were comparing apples to a different variety of apples. Indeed, it is also worth noting that culinary traditions are also seen as a form of art, possibly also contributing to these results.

The fact that participants reported higher appraisals in the case of the artworks suggests that this design indeed did elicit slightly more positive responses. On the one hand, it is important to note that, the *subjective* “art”/“not-art” label provided by the participants for their interventions (regardless of the actual assigned condition), did not result in significant differences between the impact of the cultural engagement at the level of our Wellbeing DVs, regardless of the higher appraisals. Thus, our conclusion and overall interpretation is that the label of art alone does not appear to be a necessarily important factor of cultural interventions but rather the subjective experience that the individual has with the content is much more important. This being said, these interpretation rest upon the design of the study with the inclusion of stimuli that were chosen to be similar. It would be important to further any claim based on the label of art or non-art with stimuli that are more distinct, or possibly to use a top–down manipulation of art context (see Kirk et al., 2009). We encourage further research to address participants’ subjective experience in both arts and cultural interventions rather than assuming that art by way of ‘aura’ is necessarily better.

### Potential Mechanisms for the Modulation of Mood, Anxiety, and Wellbeing

Our study also sheds light on possible potential underlying mechanisms for how art and culture can lead to wellbeing impacts. Our stepdown analysis highlighted that online cultural engagement might especially be an avenue to regulating mood, which possibly drives other positive impacts (state-anxiety, subjective wellbeing). This highlight of mood regulation is supported by past research, which has found emotion and mood regulation as a common beneficial outcome of a variety of types of arts and cultural engagement (Ivcevic and Brackett, 2015; Fancourt et al., 2019, 2021).

The significant relationships between the impacts on mood and individuals’ appraisals and cognitive-emotional experience adds new data to our understanding of how art interventions might work. The finding that higher positive cognitive-emotional states (e.g., joy, and social terms such as confident, compassion, gratitude) and more profound states such as free, moved, awe, sublime, raise interesting implications for past arguments that contributors to wellbeing impacts beyond mere pleasure. For example, many of these terms could be argued to overlap with Kaplan’s (1995) discussion of “fascination” as a pillar of restorative effects from viewing nature. Empirical evidence for such nuanced aspects in art engagement is only recently emerging (Pelowski et al., 2017c). However, it seems plausible that encouraging specific types of cognitive-emotional states, perhaps through choice of content, is an important consideration when designing interventions and is another target for further research.

Several of these states (awe, moved) are also suggested to be prototypical emotions in aesthetic experience (Fingerhut and Prinz, 2020). Along with our findings of the relationship of beauty, meaningfulness, and other positive appraisals to individual’s change in mood, one could make a claim that the present results support arguments highlighting aesthetic experiences as an important factor in art interventions (Sachs et al., 2015; Fancourt and Finn, 2019; Mastandrea et al., 2019). Fancourt and Finn (2019), include aesthetic experience in their summary of the mechanisms underlying the impact of art engagement on health and wellbeing while Mastandrea et al. (2019) further note that aesthetic experiences from visual art can induce highly pleasurable states and these states can impact affect and aid mood regulation. In future research, these features of art and cultural interventions need to be further teased apart so that we may understand what types of experiences, and which aspects of those experiences can impact which types of individuals.

### Duration and Dosage

Another interesting finding of the study involves the required time and amount of art. Unlike previous studies that have typically either installed some works of art or asked individuals to have a complete museum experience, in the present study we found improvement in wellbeing variables even with an interactive exhibition of one painting in under 5 min. This viewing duration aligns with typical ranges of individual artwork engagement in museums (Pelowski et al., 2017a). The results are also in line with other wellbeing findings. For example, the Cracknell et al. (2016) study of aquarium-viewing found that peak impacts on mood tended to be delivered within the first 5 min. Our results provide a suggestion for the possibility of “micro-dosing” art and culture that might fit into everyday routines, delivered online.

### Caveats and Targets for Future Research

This study is, of course, not without its limitations. It should, we would argue, be treated as a first exploratory step, with its findings viewed primarily as calls for future research. Readers should also take our results and interpretations with our sample size in mind. Although we found that, indeed, there is a measurable, systematic effect on a variety of variables related to wellbeing as they are self-reported by participants, it is important to consider several factors that could be raised about this result. First, in regard to the intervention itself, we had little control over what type of device was used (computer or phone), in which setting (home, office, while commuting, in hospital), and how intently participants engaged with the chosen stimuli. Similarly, in line with the nature of this type of ecological online experiment we were unable to supervise participants to see if they viewed the stimuli with a desirable amount of concentration.

We recommend that future study should therefore not only test for replication, but also include more rigorous and more controlled paradigm (e.g., using a webcam to monitor engagement, recording screen and mouse movement, etc.). That said, it could also be argued that the experience of viewing the online exhibitions, where participants could freely engage with the stimuli in their home environments, represents a strength of this study, as it reflects findings that can be generalized to more ecologically valid scenarios. Indeed, regardless of the confounding behavior that participants might have engaged in, we still found a systematic improvement in their wellbeing, suggesting that such online engagement, not in a lab where researchers ensure no distractions, but rather in the real world, is viable and deserves further study.

It is also possible due to the pre-post nature of our design that participants’ answers may have been impacted by, for example, a placebo effect or more social-driven factors such as the Hawthorne effect (Adair, 1984) in which participants might guess the study goals and respond how they feel they should (desirability effect or command bias). Although this is possible, as is the case for many psychological studies, we do not believe this precludes our results from a meaningful interpretation. Indeed, the supposed Hawthorne effect was neither consistent across all wellbeing variables (e.g., we did not find significant overall changes in positive mood or satisfaction with life) nor across all individuals (for each variable, some participants indicated that their positive mood, for example, decreased, see Figure 4). Further, due to the randomization of the six batteries of DV questions, it would be difficult for individuals to remember so many questions to then seek based on their figuring out the hypothesis to fake their answers. Thus, despite these noteworthy limitations, our results still suggest that online cultural intervention on specific facets of wellbeing is a viable area for more research. As a next step, we strongly encourage the addition of a control condition to strengthen the interpretability of the effect possibly through larger sample sizes based on these reported effect sizes, and the inclusion of a post only comparison group or other control groups.

Additionally, we are unable to determine the causality of the relationship between appraisals, cognitive-emotional experience, and impacts on mood and anxiety. Participants first viewed the cultural content, then indicated which cognitive-emotional states they experienced while viewing, and subsequently provided their appraisals of the exhibition, followed by their actual mental wellbeing state. We would encourage further research to examine if subjective experiences, such as beauty and meaningfulness, mediate the impact of said intervention on mood and anxiety. Or, alternately, if an improved cognitive, emotional, or physiological state influence reported appraisals. It would be interesting to compare these effects to other—perhaps less visually appealing or more information-focused—conditions, or stimuli that might not be spontaneously labeled as art but rather lacking any trace of intentionally of human connection.

## Conclusion

In conclusion, the results of this paper suggest that online cultural engagement, including but not limited to fine art, does seem to be a viable tool to support individuals’ mood, anxiety, loneliness and wellbeing especially when such content is beautiful, meaningful, and inspires positive cognitive-emotional states in the viewer.

## Data Availability Statement

The datasets presented in this study can be found in online repositories. The names of the repository/repositories and accession number(s) can be found below: https://github.com/giacomobignardi/Trupp-online-art-wellbeing.

## Ethics Statement

Ethical review and approval was not required for the study on human participants in accordance with the local legislation and institutional requirements. The patients/participants provided their written informed consent to participate in this study.

## Author Contributions

MT, KC, and MP contributed to the design of the study. MT and KC conducted the literature review. MT prepared the materials and collected and processed the data. MT, GB, ES, and MP contributed to the planning of the analysis. GB and MP conducted the analysis. MT and ES validated the analytical work. MT, GB, and KC drafted the manuscript. MP supervised the study. MT and MP revised the manuscript. All authors contributed to the article and approved the submitted version.

## Conflict of Interest

The authors declare that the research was conducted in the absence of any commercial or financial relationships that could be construed as a potential conflict of interest.

## Publisher’s Note

All claims expressed in this article are solely those of the authors and do not necessarily represent those of their affiliated organizations, or those of the publisher, the editors and the reviewers. Any product that may be evaluated in this article, or claim that may be made by its manufacturer, is not guaranteed or endorsed by the publisher.

## Footnotes

1 Interestingly, although one might surmise that a solution to loneliness requires being with other people or engaging at least in communal activity, one of the topics specifically addressed in Covid-19 times-related online art was loneliness, with several exhibitions featuring works on this theme (Jones, 2020; Knott, 2020).

2 Note, we are not making any ontological claim regarding the definition of art, however, we selected our stimuli so the painting was an example of objects highly popular and well-known to the general public as fine art, whereas the non-art condition had no clear connection to art and was focused on a different aspect of culture, namely the cultural/history of food.
