# Supplementary material for: Can a Brief Interaction With Online, Digital Art Improve Wellbeing? A Comparative Study of the Impact of Online Art and Culture Presentations on Mood, State-Anxiety, Subjective Wellbeing, and Loneliness
Source: Front Psychol. 2022 Jun 30;13:782033. doi: 10.3389/fpsyg.2022.782033 (PMC9281502; doi:10.3389/fpsyg.2022.782033)

Supplementary Materials Table of Contents

1. Stimuli Information. Access link and Accompanying Text of Stimuli
2. Table S1. Sample Characteristics and Lockdown Characteristics
3. Table S2. List of Emotional-Cognitive Iems in Order of in-text Figure 2a
4. Table S3. ANOVA– Condition X Block Interactions per DV
5. Table S4. Correlation between Appraisals of Experience and Change in Wellbeing DVs
6. Table S5. Appraisals of Experience split by Assigned (Water-lilies, Bento) and Reported 'Art'/ 'Non-Art' Conditions
7. Table S6. Mean Change of Wellbeing DVs and Confidence Intervals Split by Reported Condition
8. Table S7. Principal Component Analysis (Rotated) of Cognitive-Emotion List
9. Table S8. Summary of Regression Analysis for Wellbeing DVs – Combined samples
10. Table S9. Summary of Regression Analysis for Wellbeing DVs – Water-lilies Condition
11. Table S10. Summary of Regression Analysis for Wellbeing DVs – Bento Condition
12. Figure S1. Histogram of Time Spent Viewing per Condition
13. Figure S2. Wellbeing DV Correlations, Pre, Post, and Pre-Post Change
14. Figure S3. Scatterplots of Time Spent and each DV per Condition
15. Figure S4. Distributions of Appraisals

Stimuli information; Access link and accompanying text

Monet: The Water-Lily Pond. An in-painting tour from the National Gallery, London
(https://artsandculture.google.com/story/monet-the-water-lily-pond/WgIS72lKcegxJQ)

1. It wasn't actually a painting that Monet deemed his 'greatest work of art' but the beautiful gardens he created at his home in Giverny. In his later years, it became his sole subject.
2. The bridge, which Monet designed himself, shows the influence of Japanese art on his work. This is one of 18 canvases of this view in differing light conditions that Monet started in summer of 1899, the same year he started painting Waterloo and Charing Cross bridges.
3. The late afternoon sun casts a shaft of light over the bridge, illuminating the right-hand side in pale green in contrast to the prevailing darker blue-green.
4. The bold line of the bridge and the longer brushstrokes of the reeds provide a contrast to the small daubs of colour of the water-lilies.
5. Monet's water-lilies were a hybrid breed in pink and yellow as well as white.
6. The undersides of the water-lilies were dark red, the same colour in which Money signed the painting. Red is on the other side of the colour wheel to the green that dominates the painting; this contrast was in keeping with Monet's interest in complementary colours.
7. Among the mass of water-lilies, you can also see the reflection of the willow trees on the surface of the pond.

A Bitesize History of Japanese Food. Explore a bento box of mouth-watering facts about Japan's iconic cuisine
(https://artsandculture.google.com/story/vAVBze4XARcz7g)

1. Japanese food has won over the hearts (and stomachs) of people all over the world, and was even awarded the status of intangible cultural heritage by UNESCO. Here we take a tour of some of the most important moments from history that made the meals we love today.
2. Although fish and meat are an integral part of the Japanese diet today, the cuisine was actually once vegetarian! When Buddhism was introduced to Japan in the Kofun period (300-538 CE) it became forbidden to consume animals.
3. Sake, also known as nihonshu (Japanese liquor), originated in the Nara period (710-794 CE) and can be drunk either hot or cold. It's brewed using only 4 ingredients – can you guess them?
4. Rice, water yeast, and…mold – some ingredients are definitely more appetizing than others.
5. Contrary to popular belief, Japanese green tea actually originated in China before it was introduced to Japan in the 9th century. Rumor has it that it was discovered when some tea leaves fell into an Emperor's pot of hot water.
6. Where would Japanese cuisine be without rice? The grain was first cultivated in the yayoi period (1,000 BCE -300 CE) and ancient traditions, such as eating sticky cakes made from mochigome (glutinous rice) every Japanese New Year, have stuck around until today. New year, same mochi!
7. Chopsticks can be used to cook, stir, serve and eat. They were invented in the Kofun period but many people at the time still ate with their hands as only the nobility could afford these slender utensils.
8. Japanese cuisine started gaining its flavor in 17th century Edo, which later became known as Tokyo. The city is now home to the most restaurants with Michelin stars in the world.
9. The Edo period (1603-1868 CE) was also known as the samurai age. It wouldn't have been a surprise to see the streets filled with sword-swinging samurais standing next to vegetable farmers selling their produce.
10. How do you like your sushi, hand-held or squeezed? Oshizushi (squeezed sushi) was the main style of sushi in the early Edo period until nigiri (hand-held sushi) was created.

11. Ramen has always been a go-to student meal. After an influx of students moved from China to Japan in the 17th century, restaurants started to fuse Chinese noodles with Japanese cuisine to create a quick and easy dish.

12. Japanese food may have been grown in the fields but it was raised in the streets. As the Edo population grew to 1 million in the 18th century, an influx of single men brought about a new style of eating while standing at food stalls called yatai.

13. What comes to mind when you think of fast food? It might not be nigiri sushi, tempura, and soba noodles, but these were actually known as the fast-foods of the Edo era.

14. You can't have Japanese cuisine without umami – the fifth taste that combines sweet, sour, salty, and bitter. In 1908, chemist Kikunae Ikeda discovered this taste which can be found in a wide variety of foods, from peas and pork to cheese and carrots.

15. From the origins of Japan's cuisine to its influence today, the history of these mouth-watering dishes gives you a taste of where Japanese food came from.

Table S1

Sample characteristics and lockdown characteristics with random allocation tests

Variable		Total	Water-lilies	Bento	Comparison Test	Statistic	P value
Age	M (SD)	34.9 (14.5)	35.4 (14.4)	34.4 (14.6)	t test	0.30	.761
Art Interest	M (SD)	5.84 (1.44)	5.85 (1.42)	5.84 (1.48)	t test	0.23	.977
**Before lockdown how often did you….**							
Visit art museums/galleries?	Md	4.5	3	5	Wilcoxon	-0.07	.938
Read about art?	Md	4	4	3.5	Wilcoxon	-0.70	.484
Look at pictures of art?	Md	4	4	4	Wilcoxon	-0.44	.658
Attend art events?	Md	3	3	3	Wilcoxon	-0.22	.827
**Personality**							
Openness	M (SD)	5.75 (0.95)	5.61 (0.99)	5.88 (0.90)	t test	-1.27	.209
Conscientiousness	M (SD)	5.39 (1.18)	5.40 (1.20)	5.39 (1.17)	t test	0.05	.958
Extraversion	M (SD)	4.17 (1.37)	4 (1.33)	4.32 (1.40)	t test	-1.07	.289
Emotional Stability	M (SD)	4.27 (1.44)	4.26 (1.36)	4.27 (1.39)	t test	-0.03	.974
Agreeableness	M (SD)	4.88 (1.13)	4.99 (1.12)	4.78 (1.15)	t test	0.82	.413
**Pre-Wellbeing DVs**							
Neg. Mood	M (SD)	3.01 (1.43)	3.08 (1.32)	2.95 (1.53)	t test	0.09	.929
Pos. Mood	M (SD)	4.51 (1.32)	4.31 (1.39)	4.69 (1.24)	t test	-1.03	.306
Anxiety	M (SD)	3.44 (1.26)	3.60 (1.18)	3.31 (1.32)	t test	0.94	.351
Loneliness	M (SD)	3.50 (1.00)	3.49 (1.10)	3.50 (0.93)	t test	-0.31	.756
Sat. with life	M (SD)	4.53 (1.24)	4.54 (1.28)	4.52 (1.21)	t test	0.49	.961
Wellbeing	M (SD)	4.34 (1.12)	4.26 (1.12)	4.41 (1.14)	t test	0.01	.991

Variable		Total	Water-lilies	Bento	Comparison Test	Statistic	P value
Length of Lockdown	M (SD)	50.6 (16.5)	52 (15.4)	49.4 (17.5)	t test	0.73	.464
Others living in residence	Md	3	3	2	Wilcoxon	-0.98	.327
I am only staying at my house/personal ground	%	10.7	15	6.8			
I am only leaving my house for vital activities such as shopping and health services	%	31	27.5	34.1			
I am also leaving the house for social activities with people that I am not living with (in the given restrictions)	%	10.7	10	11.4			
I am also leaving the house for my work	%	9.5	2.5	15.9			
I am also leaving my house for sports or walks with the people I am living with only	%	38.1	45	31.8			
I am also leaving my house for other reasons	%		0				
I am not in lock-down at all	%		0				
					Chi square	6.55	.161

Note. Comparisons are done between the water-lilies and bento conditions to test random allocation

Table S2

Mean and confidence interval of cognitive-emotion items in order of in text Figure 2a

Cognitive-emotion item	Sample	Condition	Mean	Confidence Interval
Serenity	40	Water-lillies	4.675	.449
	44	Bento	4.136	.543
Happy	37	Water-lillies	4.486	.519
	42	Bento	4.238	.519
Stimulated	40	Water-lillies	4.45	.486
	44	Bento	4.273	.497
Harmony	40	Water-lillies	4.275	.552
	44	Bento	3.727	.584
Absorbed	40	Water-lillies	3.9	.555
	44	Bento	4	.579
Understood the intention *	37	Water-lillies	3.568	.631
	42	Bento	3.81	.643
Insight	37	Water-lillies	3.595	.596
	42	Bento	3.738	.612
Wonder	37	Water-lillies	3.757	.574
	42	Bento	3.357	.528
Self-aware	40	Water-lillies	3.65	.413
	44	Bento	3.409	.505
Hopeful	37	Water-lillies	3.73	.684
	42	Bento	3.214	.578
Relief	40	Water-lillies	3.65	.654
	44	Bento	2.955	.499
Joy	37	Water-lillies	3.081	.631
	42	Bento	3.143	.485
Light	37	Water-lillies	3.135	.677
	42	Bento	3.071	.632
Amused	37	Water-lillies	2.703	.532
	42	Bento	3.405	.576
Novelty	37	Water-lillies	2.703	.603
	42	Bento	3.405	.551
Free	37	Water-lillies	2.838	.636
	42	Bento	3.238	.599
Gratitude	37	Water-lillies	3.054	.685
	42	Bento	2.929	.521
Amazement	37	Water-lillies	2.892	.52
	42	Bento	3.071	.526
Loss of awareness of surroundings *	37	Water-lillies	2.541	.581
	42	Bento	2.976	.602
Moved	37	Water-lillies	2.811	.496
	42	Bento	2.333	.46
Thrilled	40	Water-lillies	2.45	.475
	44	Bento	2.659	.444
I changed my mind *	40	Water-lillies	2.55	.416

	44	Bento	2.386	.426
Awe	37	Water-lillies	2.703	.608
	42	Bento	2.095	.434
Confident	37	Water-lillies	2.054	.49
	42	Bento	2.714	.539
Surprise	37	Water-lillies	2.054	.49
	42	Bento	2.595	.515
Aware of my body *	37	Water-lillies	2.027	.481
	42	Bento	2.476	.525
Distracted	37	Water-lillies	2.027	.575
	42	Bento	2.167	.537
Bored	40	Water-lillies	1.9	.451
	44	Bento	2.273	.55
Epiphany	40	Water-lillies	1.975	.38
	44	Bento	2.045	.42
Euphoria	37	Water-lillies	1.919	.474
	42	Bento	1.881	.447
Profundity	37	Water-lillies	1.838	.42
	42	Bento	1.905	.471
Sublime	37	Water-lillies	2	.458
	42	Bento	1.738	.408
Transformed	37	Water-lillies	1.838	.468
	42	Bento	1.881	.42
Compassion	37	Water-lillies	1.838	.441
	42	Bento	1.857	.485
Anxiety	40	Water-lillies	1.6	.259
	44	Bento	1.932	.367
Disappointed	37	Water-lillies	1.405	.404
	42	Bento	1.714	.458
Vulnerable	37	Water-lillies	1.757	.541
	42	Bento	1.238	.274
Changed something about image of myself *	37	Water-lillies	1.432	.31
	42	Bento	1.524	.332
Overwhelmed	37	Water-lillies	1.27	.203
	42	Bento	1.5	.325
Sad	37	Water-lillies	1.541	.374
	42	Bento	1.19	.172
Confused	37	Water-lillies	1.216	.195
	42	Bento	1.5	.317
Chills	37	Water-lillies	1.432	.31
	42	Bento	1.262	.323
Stress	37	Water-lillies	1.216	.285
	42	Bento	1.405	.276
Powerless	37	Water-lillies	1.297	.367
	42	Bento	1.262	.207
I needed to leave/stop looking *	37	Water-lillies	1.081	.121
	42	Bento	1.476	.414
Shock	40	Water-lillies	1.075	.85

	44	Bento	1.455	.241
Jealous	37	Water-lillies	1.243	.346
	42	Bento	1.286	.339
Watched by others *	37	Water-lillies	1.243	.228
	42	Bento	1.286	.21
Anger	40	Water-lillies	1.075	.85
	44	Bento	1.295	.275
Like Crying *	37	Water-lillies	1.216	.295
	42	Bento	1.143	.147
Guilt	37	Water-lillies	1.162	.242
	42	Bento	1.095	.115
Disgusted	37	Water-lillies	1.081	.164
	42	Bento	1.167	.181
Embarrassed	40	Water-lillies	1.075	.85
	44	Bento	1.159	.16
Shame	37	Water-lillies	1.081	.164
	42	Bento	1.024	.48
Fear	37	Water-lillies	1.081	.121
	42	Bento	1.024	.48

Note. *name has been shortened on Figure 2a in manuscript

Table S3

Analysis of Variance – Condition X Block Interactions per DV

Variable	*df*	*F*	*p*	n_p^2	90% CI	
					LL	UL
Negative mood	1, 76	.011	.918	.00	.00	.01
Positive mood	1, 76	1.311	.256	.02	.00	.09
Anxiety	1, 76	.95	.332	.01	.00	.08
Lone	1, 76	.001	.971	.00	.00	.00
Sat. w. life	1, 76	1.912	.171	.01	.00	.07
Wellbeing	1, 76	.008	.929	.00	.00	.01

Note. CI = confidence interval; LL = lower limit; UL = upper limit.

Table S4

Correlation between experience appraisals and change in Wellbeing DVs

Appraisals	Pos. mood		Neg. mood		Anxiety		Loneliness		Wellbeing		Sat. with life	
Combined Conditions	r_s	*p*	r_s	*p*	r_s	*p*	r_s	*p*	r_s	*p*	r_s	*p*
Desire	**0.22**	**.049**	**-0.27**	**.015**	**-0.22**	**.048**	0.07	.563	-0.08	.471	0.13	.249
Meaning	0.02	.861	**-0.34**	**.002**	-0.16	.165	-0.06	.693	-0.02	.892	-0.06	.623
Beauty	0.17	.130	**-0.29**	**.009**	-0.21	.062	-0.13	.254	0.03	.791	0.05	.645
Goodness	**0.24**	**.038**	-0.22	.057	**-0.24**	**.032**	**-0.33**	**.002**	0.05	.661	0.05	.684
Water-lilies												
Desire	**0.33**	**.047**	**-0.43**	**.001**	-0.29	.087	0.08	.662	-0.17	.316	0.02	.928
Meaning	0.01	.970	**-0.36**	**.029**	-0.28	.097	0.06	.743	-0.21	.230	-0.19	.274
Beauty	0.10	.568	-0.20	.234	-0.29	.086	-0.02	.910	-0.17	.319	-0.01	.955
Goodness	0.13	.456	0.08	.629	-0.22	.201	-0.19	.274	-0.09	.603	-0.14	.421
Bento												
Desire	0.07	.648	-0.11	.468	0.18	.242	0.08	.609	-0.01	.937	0.18	.242
Meaning	-0.05	.767	**-0.32**	**.041**	-0.04	.795	-0.14	.362	0.14	.384	-0.04	.795
Beauty	0.08	.622	**-0.32**	**.038**	-0.7	.681	-0.23	.145	0.23	.137	-0.07	.681
Goodness	0.17	.273	**-0.42**	**.001**	-0.02	.878	**-0.44**	**.003**	0.16	.319	-0.02	.878

Note. Spearman correlations.

Table S5

Appraisals and DV mean changes based on assigned and reported art and non-art conditions

Variable		Assigned Condition		Reported Condition		Total [e]
		Water-lilies [a]	Bento [b]	'I saw art' [c]	'Something else' [d]	
Desire to visit again	*M* (*SD*)	4.08 (1.97)	3.30 (1.86)	3.87 (1.95)	2.81 (1.68)	3.67 (1.94)
Meaningfulness	*M* (*SD*)	4.55 (1.69)	3.68 (1.74)	4.31 (1.58)	3.19 (2.23)	4.10 (1.76)
Beauty	*M* (*SD*)	5.62 (1.53)	3.96 (1.83)	5.16 (1.57)	3.00 (2.13)	4.75 (1.88)
Goodness	*M* (*SD*)	5.92 (1.18)	4.23 (1.74)	5.50 (1.24)	3.06 (2.08)	5.04 (1.71)

Note. [a] N = 40; [b] N = 44; [c] N = 68; [d] N = 16; [e] N = 84.

Table S6

Mean change of Wellbeing DVs and confidence intervals split by reported condition

Variable	Reported Condition			
	'I saw art' [c]		'Something else' [d]	
	M	CI	M	CI
Neg. Mood	-0.33	0.24	-0.27	0.70
Pos. Mood	0.25	0.25	0.47	0.78
Anxiety	-0.30	0.20	-0.34	0.54
Loneliness	-0.20	0.12	-0.03	0.27
Sat. w Life	-0.05	0.12	0.03	0.30
Wellbeing	0.20	0.15	0.43	0.51

Note. [c] N = 68; [d] N = 16;

Table S7

Principal Component Analysis (Rotated) of Cognitive-Emotion List

Variable	PCA factor			
	1	2	3	4
confident	**0.80**	-0.18	0.09	0.06
changed something about image of myself	**0.79**	0.00	0.10	-0.13
compassion	**0.77**	-0.02	0.06	-0.12
gratitude	**0.76**	-0.05	-0.19	0.10
free	**0.73**	0.20	-0.16	-0.10
sublime	**0.72**	0.15	-0.08	-0.26
profundity	**0.72**	-0.10	-0.01	-0.13
joy	**0.69**	0.08	-0.13	0.13
light	**0.68**	0.13	-0.20	0.07
moved	**0.65**	-0.06	-0.09	0.26
euphoria	**0.62**	-0.02	0.09	0.15
awe	**0.54**	0.21	-0.28	-0.18
amazement	**0.52**	0.16	-0.39	0.17
aware body	**0.48**	0.00	0.06	0.23
wonder	**0.47**	0.33	-0.27	0.18
watched by others	**0.45**	0.11	0.27	-0.37
hopeful	**0.42**	0.25	-0.26	0.25
happy	**0.41**	0.06	-0.37	**0.38**
self-aware	**0.41**	-0.04	0.13	0.15
harmony	**0.39**	0.25	**-0.39**	0.24
novelty	**0.35**	0.06	-0.14	0.10
chills	**0.30**	0.09	0.13	0.18
fear	-0.15	**0.94**	-0.16	-0.19
like crying	-0.02	**0.87**	-0.10	-0.14
sad	0.06	**0.84**	-0.06	-0.00
powerless	-0.03	**0.78**	-0.04	-0.13
stress	-0.08	**0.72**	0.25	-0.15
shame	-0.08	**0.64**	0.23	0.20
guilt	-0.03	**0.64**	0.28	0.20
vulnerable	0.24	**0.63**	0.04	0.07
jealous	0.03	**0.46**	-0.04	0.14
distracted	0.09	**0.39**	0.26	-0.06
disappointed	0.04	0.06	**0.73**	-0.05
bored	-0.11	-0.02	**0.71**	-0.29
need to leave	0.15	0.01	**0.58**	-0.30
embarrassed	0.03	0.18	**0.56**	0.32
confused	0.05	0.43	**0.56**	0.00
serenity	0.31	0.07	**-0.54**	0.26

stimulated	0.08	0.26	**-0.52**	0.51
anger	0.04	0.07	**0.50**	0.02
anxiety	-0.10	0.15	**0.43**	0.26
relief	0.37	-0.03	**-0.41**	0.08
disgusted	-0.06	-0.07	**0.35**	0.08
absorbed	0.03	0.15	-0.37	**0.67**
thrilled	0.30	-0.03	-0.12	**0.60**
insight	0.32	-0.07	-0.13	**0.55**
change mind about meaning	-0.01	0.26	-0.09	**0.54**
epiphany	0.33	-0.07	0.17	**0.53**
amused	0.23	-0.10	-0.11	**0.51**
transformed	0.34	0.08	0.10	**0.45**
loss of awareness of surroundings	0.42	0.01	0.07	**0.44**
shock	-0.11	-0.03	0.14	**0.39**
surprised	0.27	-0.17	-0.09	**0.36**
understood the intention	0.26	-0.08	-0.05	**0.35**
overwhelmed	-0.01	0.04	0.30	**0.33**

Note. Bold values indicate loading items.

Table S8

Summary of Regression Analysis for Wellbeing DVs – Combined samples

Variable		*B*	SE_B	β	*t*	Sig. (*p*)
SAT change	constant	-.008	.054		-.151	.881
	1	.025	.059	.054	.426	.671
	2	.092	.056	.199	1.636	.106
	3	-.028	.056	-.510	-.510	.612
	4	-.038	.057	-.663	-.663	.510
Wellbeing change	constant	.257	.079		3.262	.002
	1	.156	.086	.226	1.808	.075
	2	-.156	.082	-.227	-1.908	.061
	3	-.031	.082	-.045	-.377	.707
	4	-.074	.083	-.108	-.897	.373
Pos mood change	constant	.338	.124		2.723	.008
	1	.182	.136	.169	1.340	.185
	2	-.132	.129	-.123	-1.025	.309
	3	-.167	.129	-.155	-1.300	.198
	4	-.081	.131	-.075	-.620	.537
Lone change	constant	-.171	.056		-3.049	.003
	1	-.072	.062	-.150	-1.167	.247
	2	-.048	.058	-.100	-.822	.414
	3	-.010	.058	-.020	-.165	.870
	4	.006	.059	.013	.106	.916
STAI change	constant	-.327	.094		-3.475	.001
	1	-.246	.103	-.295	-2.391	**.020**
	2	.149	.098	.178	1.519	.133
	3	.040	.097	.047	.407	.685
	4	-.049	.099	-.059	-.497	.621
Neg mood change	constant	-.324	.106		-3.061	.003
	1	-.250	.116	-.241	-2.151	**.035**
	2	.148	.110	.143	1.342	.184
	3	.412	.110	.398	3.752	**.000**
	4	.010	.112	.009	.087	.931

Note. *B* = unstandardized regression coefficient; SE_B = standard error of the coefficient; β = standardized coefficient. Bold values indicate significant p values, uncorrected.

Table S9

Summary of Regression Analysis for Wellbeing DVs – Water-lilies Condition

Variable		*B*	SE_B	β	*t*	Sig. (*p*)
Sat. life change	constant	.017	.075		.223	.825
	1	-.070	.088	-.146	-.800	.430
	2	.139	.056	.419	2.482	**.019**
	3	-.128	.089	-.241	-1.434	.162
	4	.045	.088	.091	.511	.613
Wellbeing change	constant	.247	.113		2.182	.037
	1	.283	.134	.395	2.113	**.043**
	2	-.121	.085	-.245	-1.414	.168
	3	.017	.136	.021	.122	.904
	4	-.228	.134	-.311	-1.699	.100
Pos mood change	constant	.437	.187		2.332	.027
	1	.222	.221	.194	1.003	.324
	2	-.090	.141	-.114	-.636	.530
	3	-.244	.224	-.195	-1.088	.285
	4	.132	.222	.113	.596	.556
Lone change	constant	-.211	.090		-2.349	.026
	1	-.071	.106	-.125	-.675	.505
	2	-.098	.068	-.251	-1.452	.157
	3	-.221	.107	-.352	-2.054	**.049**
	4	.039	.106	.067	.369	.714
Anxiety change	constant	-.423	.137		-3.094	.004
	1	-.428	.161	-.446	-2.649	**.013**
	2	.154	.103	.234	1.496	.145
	3	.093	.164	.089	.571	.572
	4	-.190	.162	-.194	-1.175	.250
Neg mood change	constant	-.208	.144		-1.443	.160
	1	-.369	.170	-.363	-2.166	**.039**
	2	.084	.109	.121	.777	.444
	3	.466	.173	.417	2.693	**.012**
	4	.059	.171	.057	.346	.732

Note. *B* = unstandardized regression coefficient; SE_B = standard error of the coefficient; β = standardized coefficient. Bold values indicate significant p values, uncorrected.

Table S10

Summary of Regression Analysis for Wellbeing DVs – Bento Condition

Variable		*B*	SE_B	β	*t*	Sig. (*p*)
Sat. life change	constant	-.154	.085		-1.822	.077
	1	.149	.080	.338	1.864	.071
	2	-.331	.169	-.440	-1.959	.058
	3	.174	.091	.395	1.916	.064
	4	.063	.085	.144	.747	.460
Wellbeing change	constant	.154	.128		1.202	.237
	1	.178	.121	.264	1.476	.149
	2	-.616	.255	-.534	-2.413	**.021**
	3	.118	.137	.174	.858	.396
	4	.122	.128	.182	.956	.346
Pos mood change	constant	.197	.204		.964	.342
	1	.193	.192	.188	1.006	.321
	2	-.402	.407	-.229	-.989	.330
	3	-.037	.218	-.036	-.170	.866
	4	-.091	.204	-.089	-.447	.658
Lone change	constant	-.169	.083		-2.045	.048
	1	-.097	.078	-.233	-1.247	.221
	2	.106	.165	.148	.641	.526
	3	.042	.089	.100	.473	.639
	4	-.024	.083	-.057	-.289	.774
Anxiety change	constant	-.250	.154		-1.627	.113
	1	-.139	.145	-.185	-.961	.343
	2	.155	.306	.120	.505	.617
	3	.008	.165	.010	.047	.963
	4	.006	.153	.008	.041	.968
Neg mood change	constant	-.309	.183		-1.688	.100
	1	-.252	.173	-.241	-1.463	.152
	2	.585	.365	.327	1.602	.118
	3	.276	.196	.263	1.406	.169
	4	-.121	.183	-.116	-.661	.513

Note. *B* = unstandardized regression coefficient; SE_B = standard error of the coefficient; β = standardized coefficient. Bold values indicate significant p values, uncorrected.

Figure S1

Histogram of time spent viewing per condition with medians shown in dashed lines

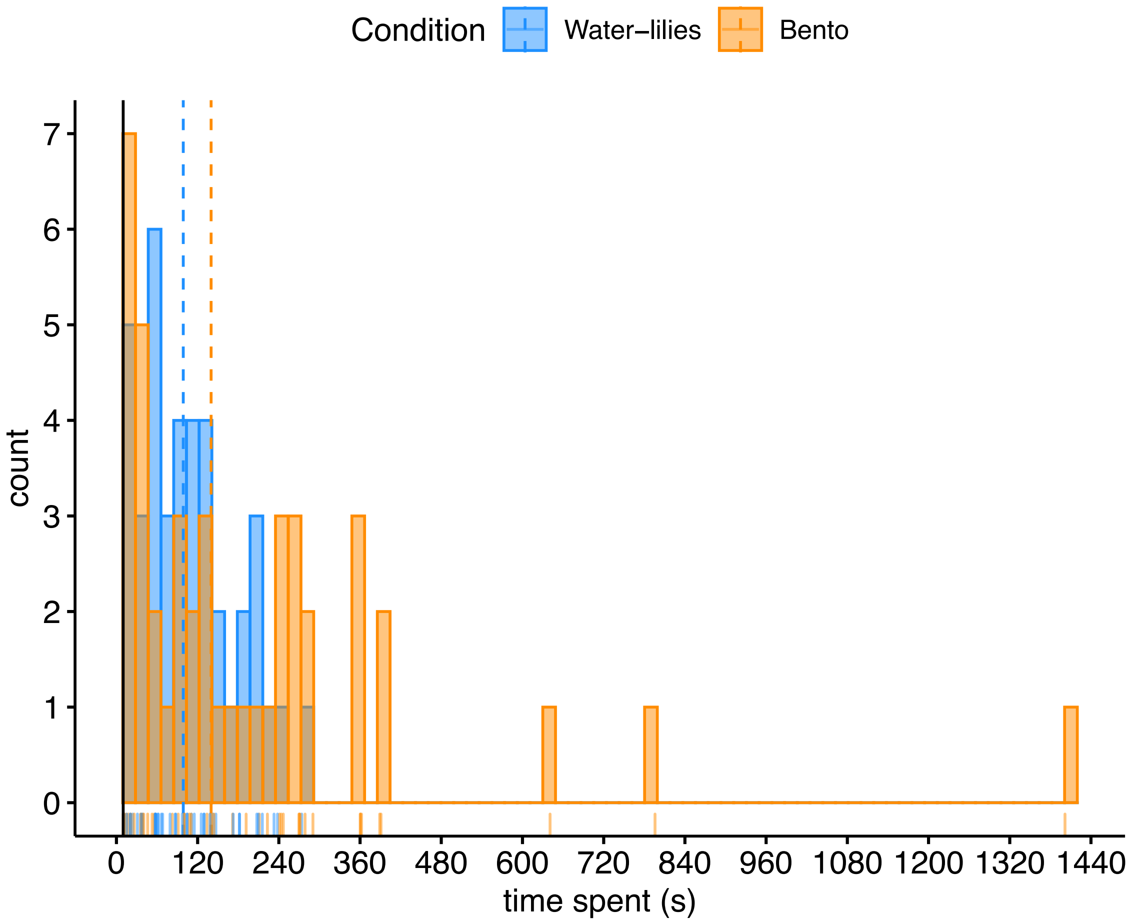

Figure S2

Correlations between pre (A), post (B), and pre-post change scores (C) for wellbeing DVs

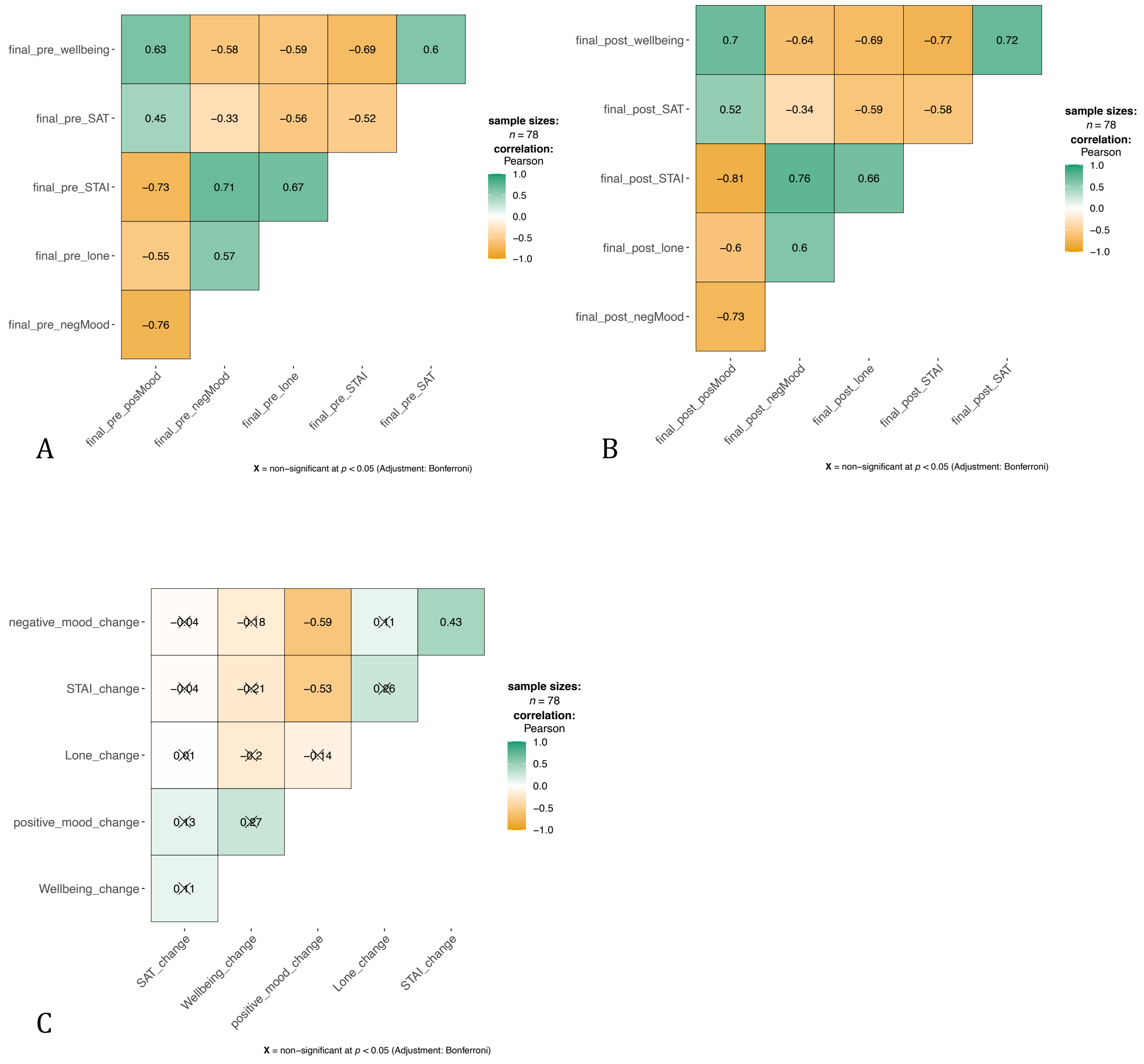

Figure S3

Scatterplots of time spent and each DV per condition with 10 second cut off marked with dashed line

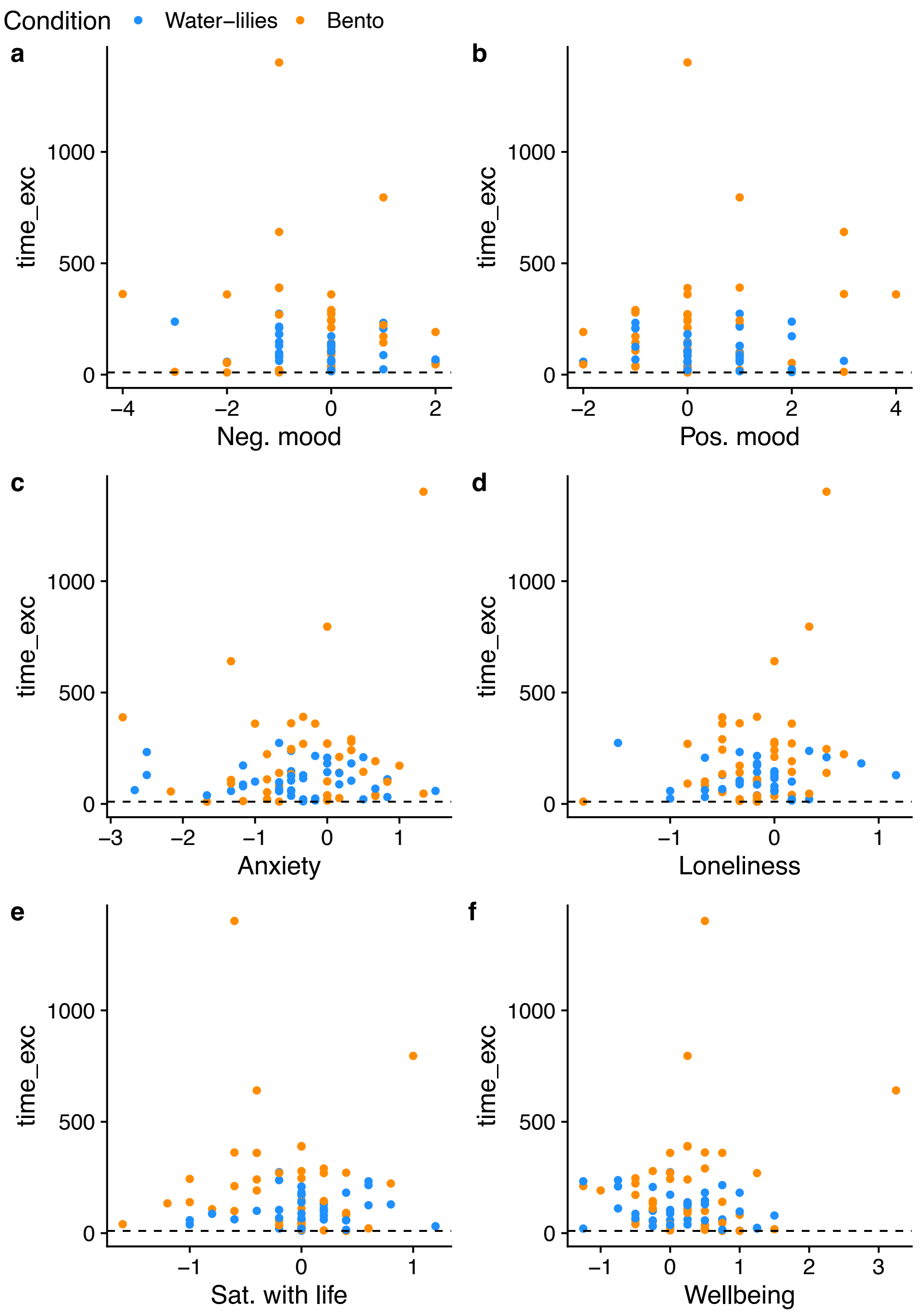

Figure S4

Distributions of Appraisals

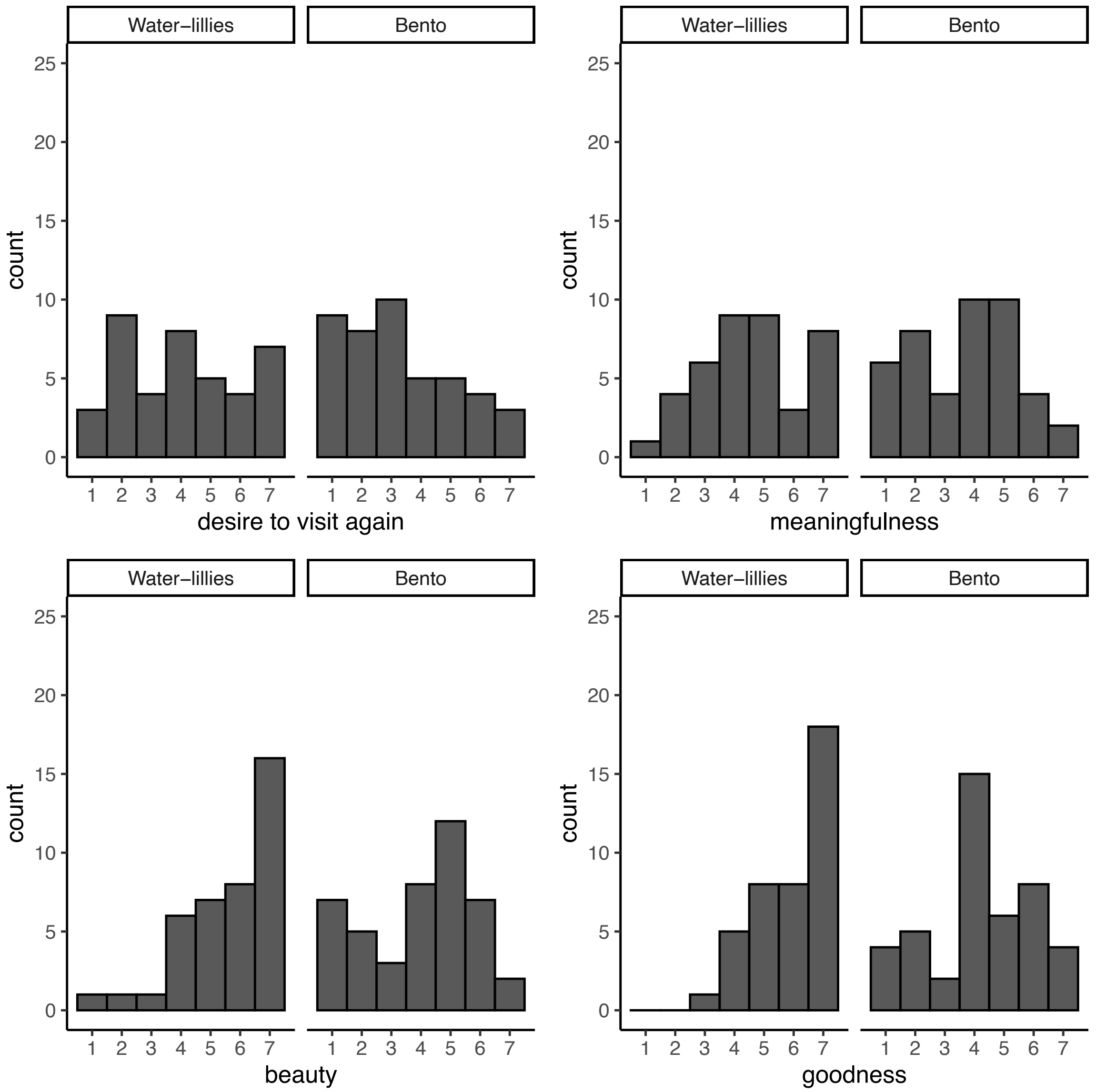

Supplement: Supplementary file 1 [file Data_Sheet_1.PDF]
